# Abiotic stressors impact outer membrane vesicle composition in a beneficial rhizobacterium: Raman spectroscopy characterization

**DOI:** 10.1038/s41598-020-78357-4

**Published:** 2020-12-04

**Authors:** Matthew Potter, Cynthia Hanson, Anne J. Anderson, Elizabeth Vargis, David W. Britt

**Affiliations:** Department of Biological Engineering, Utah State University, Logan, UT 84322 USA

**Keywords:** Biological techniques, Biophysics, Biotechnology, Cell biology, Microbiology, Engineering, Optics and photonics

## Abstract

Outer membrane vesicles (OMVs) produced by Gram-negative bacteria have roles in cell-to-cell signaling, biofilm formation, and stress responses. Here, the effects of abiotic stressors on OMV contents and composition from biofilm cells of the plant health-promoting bacterium *Pseudomonas chlororaphis* O6 (*Pc*O6) are examined. Two stressors relevant to this root-colonizing bacterium were examined: CuO nanoparticles (NPs)-a potential fertilizer and fungicide- and H_2_O_2_-released from roots during plant stress responses. Atomic force microscopy revealed 40–300 nm diameter OMVs from control and stressed biofilm cells. Raman spectroscopy with linear discriminant analysis (LDA) was used to identify changes in chemical profiles of *Pc*O6 cells and resultant OMVs according to the cellular stressor with 84.7% and 83.3% accuracies, respectively. All OMVs had higher relative concentrations of proteins, lipids, and nucleic acids than *Pc*O6 cells. The nucleic acid concentration in OMVs exhibited a cellular stressor-dependent increase: CuO NP-induced OMVs > H_2_O_2_-induced OMVs > control OMVs. Biochemical assays confirmed the presence of lipopolysaccharides, nucleic acids, and protein in OMVs; however, these assays did not discriminate OMV composition according to the cellular stressor. These results demonstrate the sensitivity of Raman spectroscopy using LDA to characterize and distinguish cellular stress effects on OMVs composition and contents.

## Introduction

Outer membrane vesicles (OMVs) are extracellular vesicles produced by Gram-negative bacteria, ranging from 20–300 nm in diameter^1–3^. OMV composition includes numerous outer membrane and periplasmic components such as proteins, lipopolysaccharide (LPS), enzymes, and in the case of pathogens, toxins^4^. OMVs may also contain DNA^5,6^ or RNA^7^. Consequently, OMV formation and release exhibit many unique functions including acting as a protein secretion system^8^, delivering virulence factors by pathogenic bacteria^9–11^, and signaling between bacteria^12,13^ and cross-kingdom with eukaryotic cells^9,13,14^. OMVs are released during all stages of cell growth in liquid and solid culture^15^. OMVs are released in biofilms and are a part of the extracellular matrix in naturally-occurring and laboratory-grown biofilms^16^. OMVs may mediate biofilm formation^17^. For several *Helicobacter pylori* strains, OMV production correlates with the ability to form and maintain the biofilm^18^. The quorum-sensing molecule 2-heptyl-3-hydroxy-4-quinolone, used for communication during biofilm formation by *Pseudomonas aeruginosa*, is present within *P. aeruginosa*-produced OMVs^19^ and is required for OMV formation by this bacterium^20^.

Although OMVs are continually released, OMV production increases in response to stress^21,22^, possibly as a mechanism to quickly release misfolded periplasmic proteins^22,23^. OMV production under stress also appears to have defensive roles. OMVs are decoys for bacteriophages and other membrane-binding antimicrobial agents thus distancing their potential to impact the live cell^24^. OMVs may contain specific enzymes and other compounds to increase bacterial survival^1,25,26^. For example, exposure to reactive oxygen species (ROS), such as H_2_O_2_, increases OMV release in several bacteria^21,27,28^. However, *H. pylori* release catalase-containing OMVs in response to ROS stress thus protecting cells from oxidative damage^28^. Another example is packaging β-lactamase into OMVs by *Moraxella catarrhalis* to inactivate β-lactam antibiotics^29^.

Virulence and signaling through OMVs released by human pathogens^11,30^ and plant pathogens^31,32^ have been investigated for managing infections. In contrast, factors stimulating OMV release and OMV roles for plant health-promoting bacteria are generally unknown. The beneficial relationship between the plant and its associated microbiome requires cross-kingdom signaling that is mediated through many small metabolites, including hormones and quorum sensing molecules^33,34^. OMV release presents one potential method for the secretion and delivery of these signals.

The potential packaging of nucleic acids and LPS into OMVs may be important as signals to the plant which then activates plant defenses. LPS is a microbe-associated molecular pattern (MAMP) and DNA fragments are damage-associated molecular patterns (DAMPs) recognized by specific plant cell receptors to trigger innate immunity^35^. OMVs contain large quantities of LPS^36^, likely due to the high surface area of these nano-vesicles. OMVs released by *P. aeruginosa* may contain plasmid DNA^12^, coding chromosomal DNA^5^, or noncoding extracellular DNA (eDNA)^37^.

Here, OMVs of a plant health-promoting bacterium, *Pseudomonas chlororaphis* O6 (*Pc*O6), were studied. *Pc*O6 is a Gram-negative, rod-shaped bacterium originally isolated from roots of commercial dryland wheat grown in Cache Valley, UT, USA^38^. *Pc*O6 is representative of many beneficial soil bacteria, boosting plant health through multiple protective pathways. *Pc*O6 is an aggressive root colonizer and forms robust biofilms on plant roots^39,40^ and abiotic surfaces^41^. Cell growth within the biofilm is nurtured through catabolism of the metabolites in the root exudates^41^. In return, the bacterium protects the host by producing phenazines and other antibacterial and antifungal compounds as well as triggering systemic resistance^42^. *Pc*O6 protects plants from drought, in part by producing a volatile, butanediol, that triggers partial stomatal closure^43^. Biofilm formation may also promote crop drought tolerance because the biofilm matrix maintains moisture around the plant roots^44,45^.

Potential OMVs are observed in atomic force microscopy images of *Pc*O6 cells^46,47^, but OMV composition and their role in *Pc*O6 signaling, biofilm architecture, and stress responses are currently unknown. Two relevant stressors to the rhizosphere, the space around plant roots, and their effects on *Pc*O6 and subsequent OMV production were examined in this study. The first stressor was CuO nanoparticles (NPs). Numerous engineered metal and metal oxide NPs are explored for agricultural applications including delivering macronutrient and micronutrient to crops^48,49^, controlling pests and pathogens^49^, and protecting crops against abiotic stresses^50^ such as drought^40,49,51,52^. In some cases, NPs enhance microbial synthesis of products that improve plant health^53^.

In *Pc*O6-colonized wheat, CuO NPs upregulate genes associated with drought tolerance^52^ and increase lignification in wheat sclerenchyma, a strengthening tissue of the plant^40^. Thus NP-induced gene expression can contribute to crop stress tolerance^52,54^. NPs also change gene expression in various beneficial and antagonistic microorganisms^54^. Sublethal doses of CuO NPs increase *Pc*O6 cell size without impeding biofilm formation^41^, decrease the production of the Fe-scavenging siderophore pyoverdine^55^, and increase the production of the plant growth regulator indole-3-acetic acid^56^. Hydrogen peroxide (H_2_O_2_) was examined as a second bacterial stressor as ROS are generated as a stress response by plant root cells^57^, including roots exposed to sublethal CuO NP challenges^58^.

To characterize OMVs, as well as any compositional changes due to these abiotic stressors, Raman spectroscopy was chosen as the primary analytical technique. Compared to other spectroscopy methods, Raman spectroscopy is well suited for biological samples as it requires minimal sample preparation, is non-destructive, and yields a linear correlation of compound concentration to signal strength^59^. Raman spectroscopy is regularly used for cellular identification and characterization, even at a single-cell level^60–62^. Changes in biomolecular profiles according to the growth stage can be detected by Raman spectroscopy in mycobacterial cells^63^. Raman spectroscopy has been used to characterize extracellular vesicles from eukaryotic cells^64–66^, but no current literature reports Raman spectroscopy characterization of OMVs.

Raman spectroscopy was used to explore the chemical signatures of biofilm *Pc*O6 cells under baseline conditions and after exposure to sublethal doses of CuO NP or H_2_O_2_. OMVs were isolated and characterized from both control and stressed cells. Spectra were compared using linear discriminant analysis (LDA). This machine learning technique creates an algorithm to sort input data sets according to treatments, then uses the algorithm to predict the treatments of the input data sets. The actual treatment and predicted treatment are then compared to determine consistencies and variations between spectra. Biochemical assays detecting protein, LPS, and DNA concentrations were performed to measure their presence in OMVs and to further examine changes in OMV composition.

## Results

### Physical characterization of outer membrane vesicles

**Figure 1:**
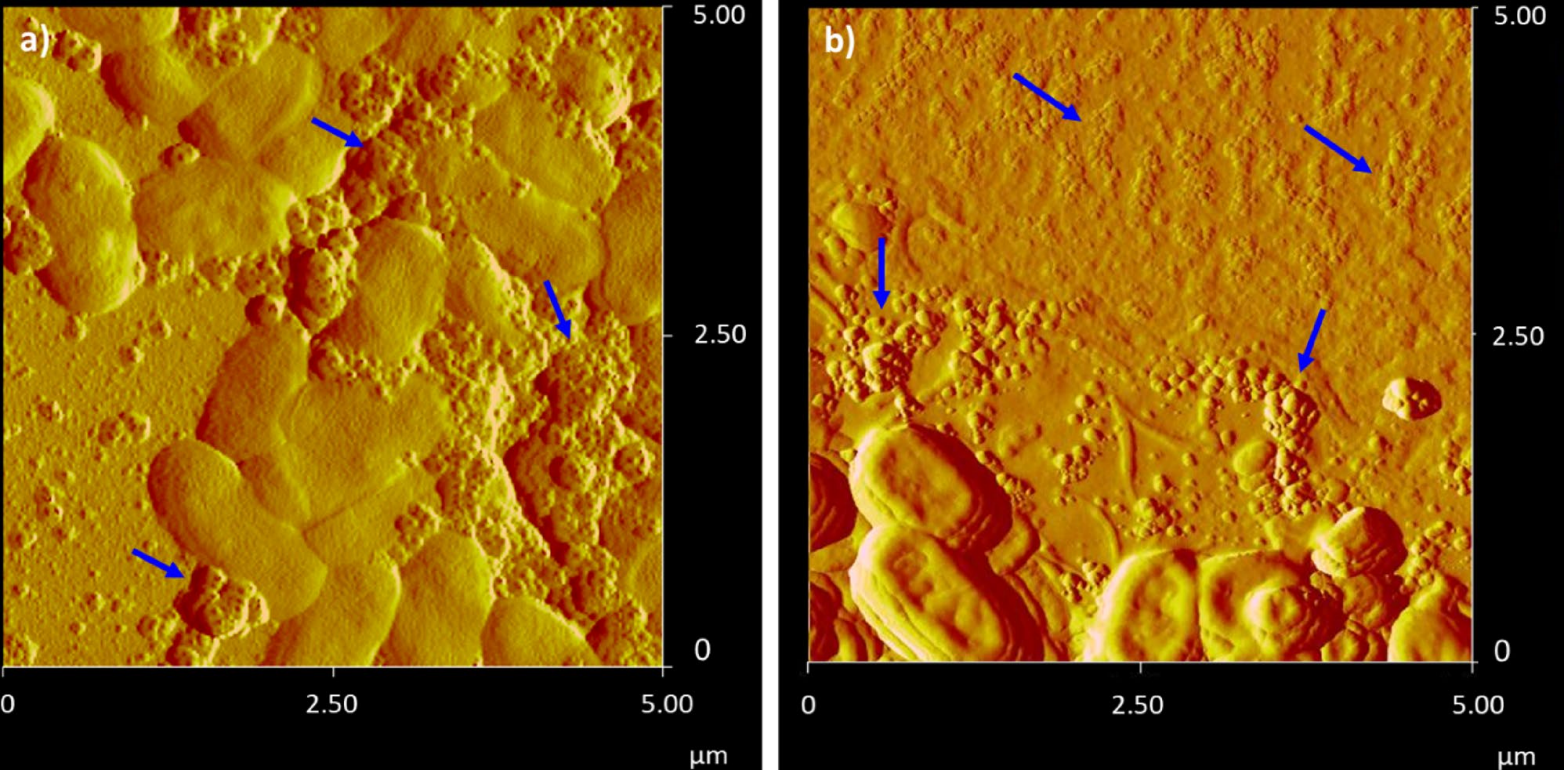
AFM amplitude images of cells from the edges of a *Pc*O6 cell smear from a biofilm grown on a 2% agar minimal medium plate onto a clean glass slide. OMVs are visible in both images as (**a**) aggregates between *Pc*O6 cells and (**b**) as linear assemblies. Arrows point to examples of these OMV aggregates.

**Figure 2:**
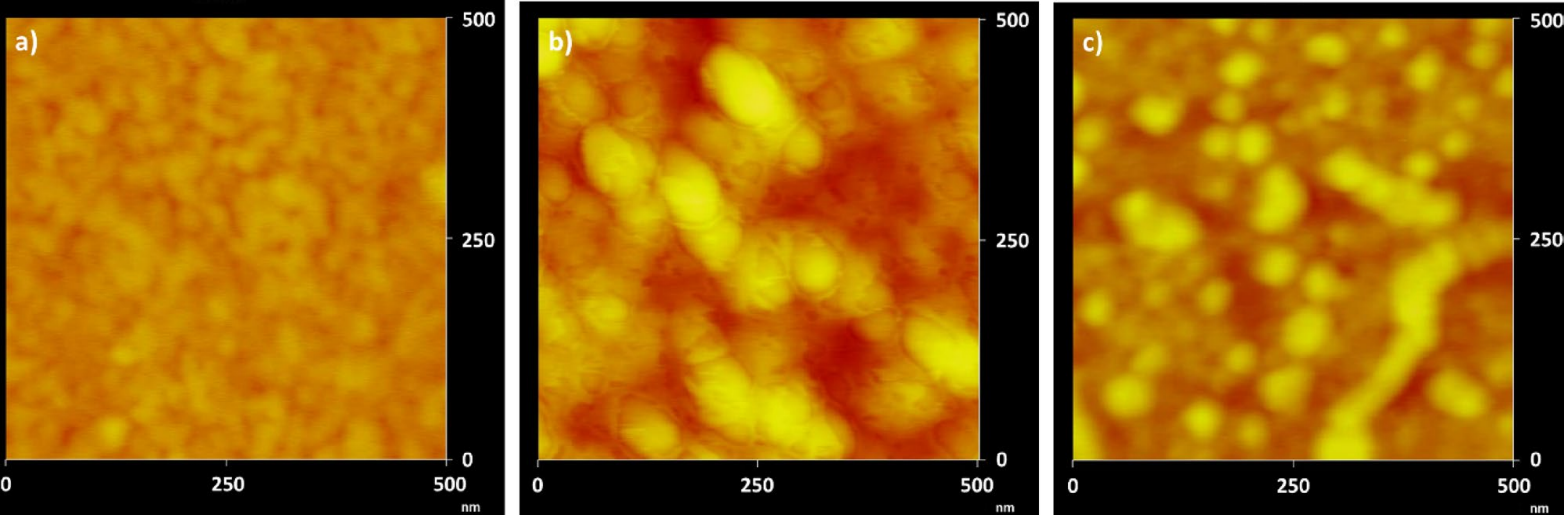
AFM height images of purified OMVs. Z-scales are from red to yellow from 0 to 25 nm, respectively. (**a**) OMVs harvested from *Pc*O6 without any stressors. (**b**) OMVs harvested from *Pc*O6 under H_2_O_2_ stress (3% v/v). (**c**) OMVs harvested from *Pc*O6 under CuO NP stress (30 mg Cu/L). Additional AFM images shown in Supplemental Fig. S1.

**Figure 3:**
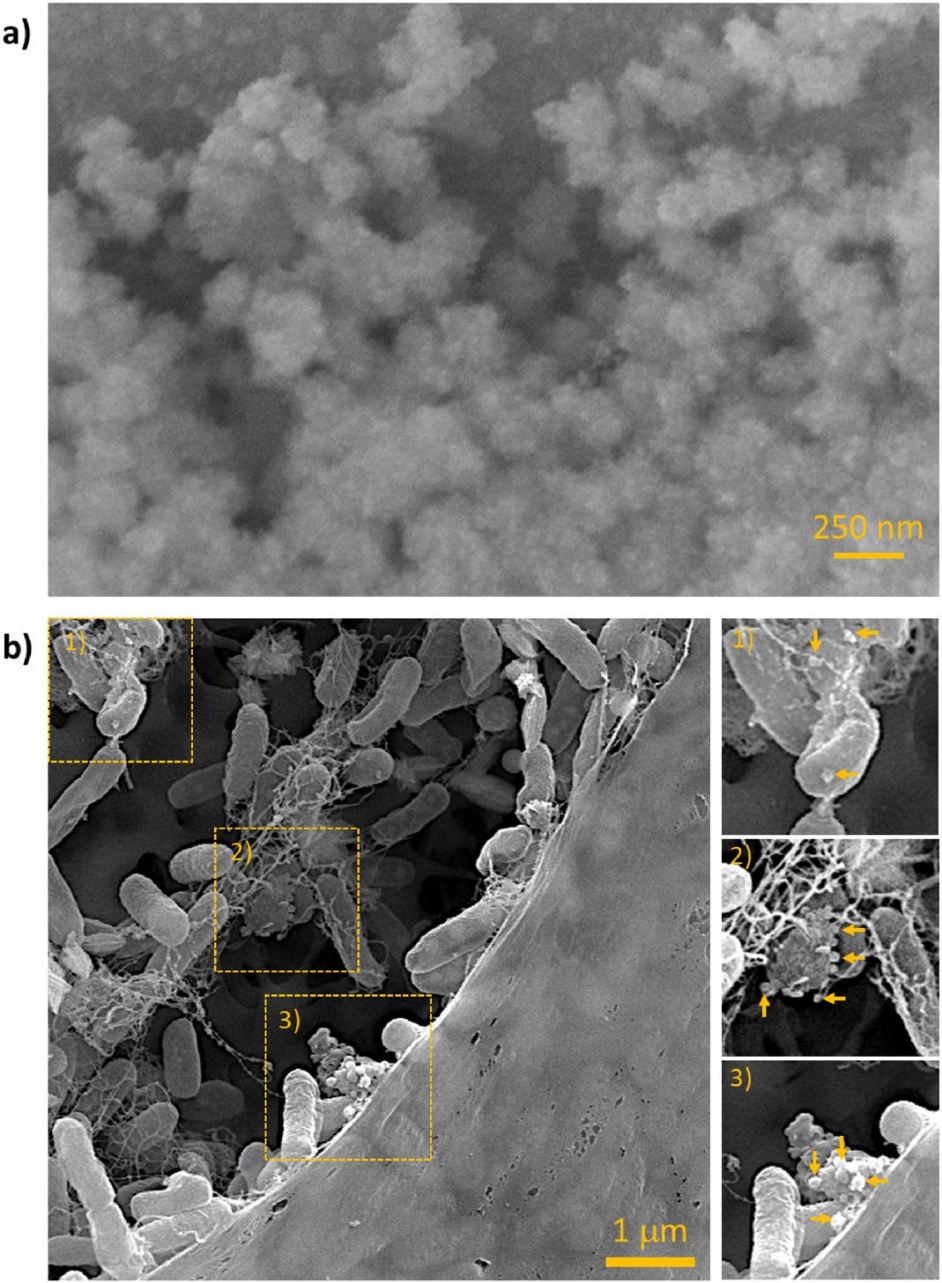
SEM images of *Pc*O6 cells and purified OMVs. (**a**) SEM image showing OMV aggregation of samples purified from *Pc*O6 biofilms grown on minimal medium plates without abiotic stressors after glutaraldehyde fixation and ethanol dehydration. (**b**) SEM image showing the cross-section of a *Pc*O6 biofilm grown on a hollow fiber membrane draped in minimal medium without any added stressors. Three areas of this image are highlighted to show potential OMVs which are marked with arrows.

OMV production by *Pc*O6 biofilm cells was confirmed in situ with imaging by atomic force microscopy (AFM) and scanning electron microscopy (SEM). AFM images of *Pc*O6 biofilm cells, transferred from minimal medium agar plates, without stressors show OMVs as aggregates between *Pc*O6 cells (Fig. 1a) and as linear assemblies apart from the cells (Fig. 1b). Purified control and stress-induced OMVs appear as clustered and linear aggregates in AFM images (Fig. 2). SEM analysis also reveals a propensity of purified OMVs to aggregate (Fig. 3). SEM images of *Pc*O6 biofilms formed on hollow fiber membranes draped in minimal medium, a substrate that allows imaging of intact biofilms encased in extracellular polymeric substances, also showed OMVs as part of the biofilm matrix as well as budding from and/or adhered to cells (Fig. 3b).

Size analysis of the OMVs in the AFM images (n = 100 from four images per treatment) showed average diameters of individual OMVs in the range of 40–190 nm, 45–295 nm, and 45–140 nm for control, CuO NP-induced, and H_2_O_2_-induced OMVs (Supplemental Fig. S2). Two-way ANOVA of the data, accounting for the treatment and the image used, showed statistically significant differences (p-value < 0.05) between several images within each treatment (data not shown) leaving uncertainty for the results comparing OMVs from one treatment. Furthermore, OMV heights in AFM images were a fraction of the OMV width, only 5–15 nm (data not shown), implying that the OMVs flattened during sample desiccation and these diameters may not represent true in vivo OMV size. These size ranges and this potential vesicle flattening has been observed by other researchers imaging vesicles with AFM^67^.

Both AFM and SEM images revealed agglomerate structures comprised of individual OMVs with clearly defined boundaries between vesicles. This propensity for OMV aggregation was confirmed with dynamic light scattering (DLS) which revealed hydrodynamic diameters ranging from 40–300 nm (Supplemental Fig. S3). The imaging and DLS results are consistent with literature reports of OMV diameters between 10 and 300 nm^11, 68^.

### Chemical characterization of *Pc*O6 cells with Raman spectroscopy

**Figure 4:**
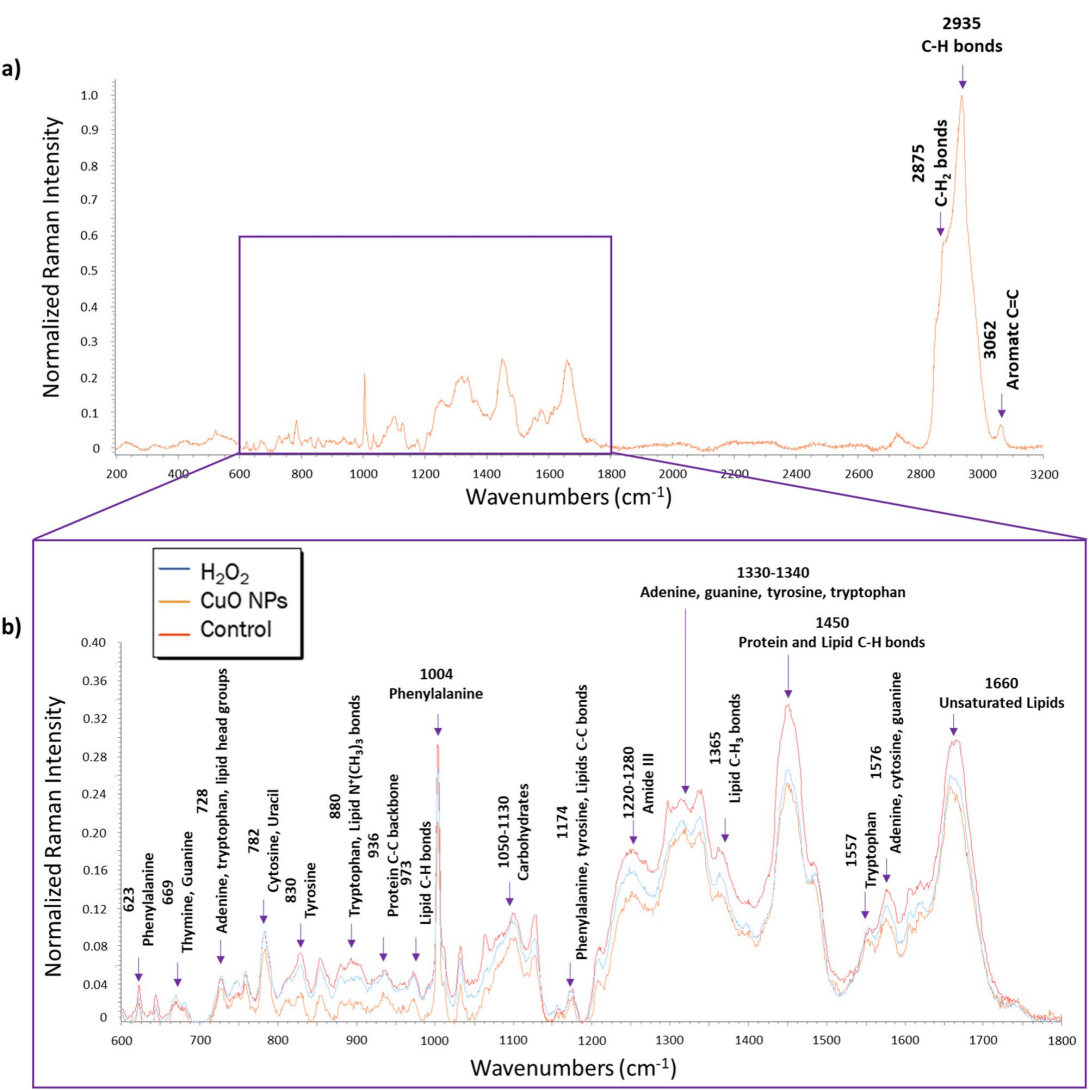
Averaged Raman spectra of intact *Pc*O6 cells (6 replicates with 4 spectra each for a total of n = 24). (**a**) Averaged Raman spectra of *Pc*O6 cells without any applied stressor from 600 to 3200 cm^−1^. No visible differences are seen in the relative peak intensities above 1800 cm^−1^ of *Pc*O6 cells of each treatment so spectra of CuO NP and H_2_O_2_ treated cells are not shown. (**b**) Averaged Raman spectra of *Pc*O6 cells treated with H_2_O_2_ stress, CuO NP stress, and a negative control with no added stressor from 600 to 1800 cm^−1^. In this region, differences are seen in relative peak intensity indicating differences in relative concentrations of various compounds. In both graphs, notable peak assignments are shown with an arrow and labeled with both the peak number and peak assignment. See Supplemental Table S1 for a list of all peak assignments and respective sources.

Raman spectroscopy was coupled with LDA to detect differences in the chemical profiles of control and stressed *Pc*O6 cells. Raman spectra of whole biofilm *Pc*O6 cells (n = 24 from 6 replicates), normalized to the highest peak of 2935 cm^−1^ (C–H bonds and lipid C–H_3_ bonds), from each treatment are shown in Fig. 4. The 2935 cm^−1^ peak was selected for normalization as it correlates to C–H bonds from all biological molecules and lipid C–H_3_ bonds. Both of these bonds are plentiful in phospholipids that are present in OMV membranes, making this peak ideal for normalization.

Peak assignments were consistent with proteins containing amino acids such as phenylalanine and tyrosine as well as unsaturated lipids and polysaccharides. The signatures of bases adenine, uracil, and guanine indicate the presence of nucleic acids. A full list of peak assignments is shown in Supplemental Table S1. There were no peaks unique for any treatment nor any peaks that disappeared in any one treatment. However, peak intensities differed between treatments with the spectra of control *Pc*O6 giving the highest relative intensities, followed by H_2_O_2_-treated *Pc*O6, then CuO NP-treated *Pc*O6. The following peaks showed the greatest decreases in peak intensity for stressed cells relative to the control: (a) 880 cm^−1^: tryptophan and lipid N^+^(CH_3_)_3_ bonds; (b) 1220–1280 cm^−1^: amide III; (c) 1330–1340 cm^−1^: adenine, guanine, tyrosine, and tryptophan; (d) 1365 cm^−1^: lipid C–H bonds; (e) 1450 cm^−1^: protein and lipid C–H bonds; and f) 1660 cm^−1^: unsaturated lipids.

**Figure 5:**
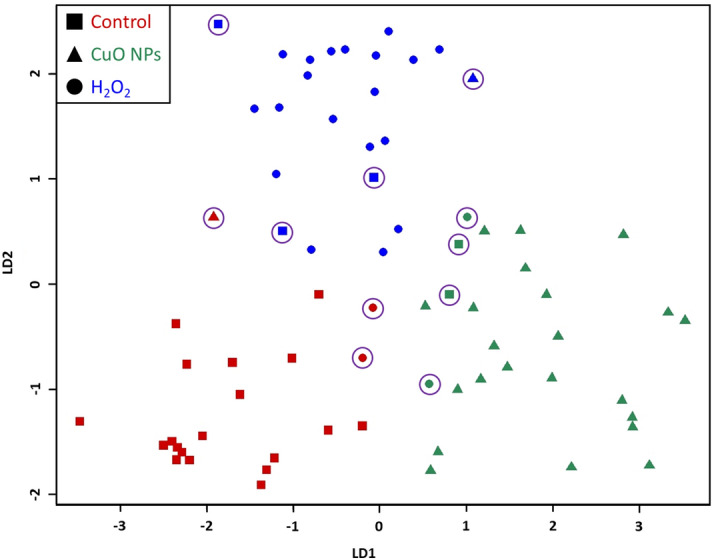
LDA plot of Raman spectra from *Pc*O6 cells. Data were truncated to 750–1700 cm^−1^ and 2670–3100 cm^−1^, where most peaks are located. The shape of the points of the plot indicate the true treatment of each spectrum whereas the color of the point indicates the predicted treatment according to LDA. Misclassified spectra are circled in purple.

Examining the majority of the Raman spectra (750–1700 and 2670–3100 cm^−1^) with LDA, it is apparent that *Pc*O6 cells exhibit distinct chemical profiles according to the applied stressor (LDA plot shown in Figure 5 and LDA confusion matrix are shown in Table 1). Though several spectra are misclassified from each treatment, the algorithm had an 84.7% accuracy. LDA was also performed on narrower regions of the Raman spectra (Supplemental Fig. S4) to correlate how restricted input data sets influenced LDA grouping and accuracy. LDA results of spectra subsets trended towards increased distance between data groupings. Accuracy of the LDA assignment varied between spectral regions.

**Table 1:** Confusion matrix showing LDA results of Raman spectra from *Pc*O6 cells.

Confusion matrix of Raman spectra of *Pc*O6 cells				
		Predicted treatment		
		Control	CuO NPs	H_2_O_2_
True treatment	Control	19	2	3
	CuO NPs	1	22	1
	H_2_O_2_	2	2	20

Data were truncated to 750–1700 cm^−1^ and 2670–3100 cm^−1^, where most peaks are located. LDA of the Raman spectra gave an 84.7% accuracy indicating that spectra of *Pc*O6 cells are highly dependent on the stressor.

The LDA plot of the 2670–3100 cm^−1^ showed loose grouping of the treatments and the lowest accuracy of the examined regions was 82% (Supplemental Fig. S4b). This region of the spectra corresponds with several C–H and C–H_2_ signatures which would be unlikely to change across experimental groups. This region also contains the 2935 cm^−1^ peak (C–H bonds) which was the highest peak in all spectra and used to normalize the spectra. By contrast, LDA of lower wavenumbers tended to show tighter and more distinct groupings and higher accuracy. This is especially notable in the 1400–1500 cm^−1^ region (Supplemental Fig. S4f) and the 1500–1700 cm^−1^ region (Supplemental Fig. S4g) which both gave 94.4% accuracies. All treatments are distinctly grouped in these LDA plots, especially the H_2_O_2_ treatment in the 1400–1500 cm^−1^ region and the CuO NP treatment in the 1500–1700 cm^−1^ region. The 1400–1500 cm^−1^ region contains several protein signatures. The 1500–1700 cm^−1^ region contains various amino acid, nucleic acid, amide, and lipid signatures (see Supplemental Table S1 for a list of all peak assignments).

### Comparing Raman spectra of *Pc*O6 cells and isolated OMVs

**Figure 6:**
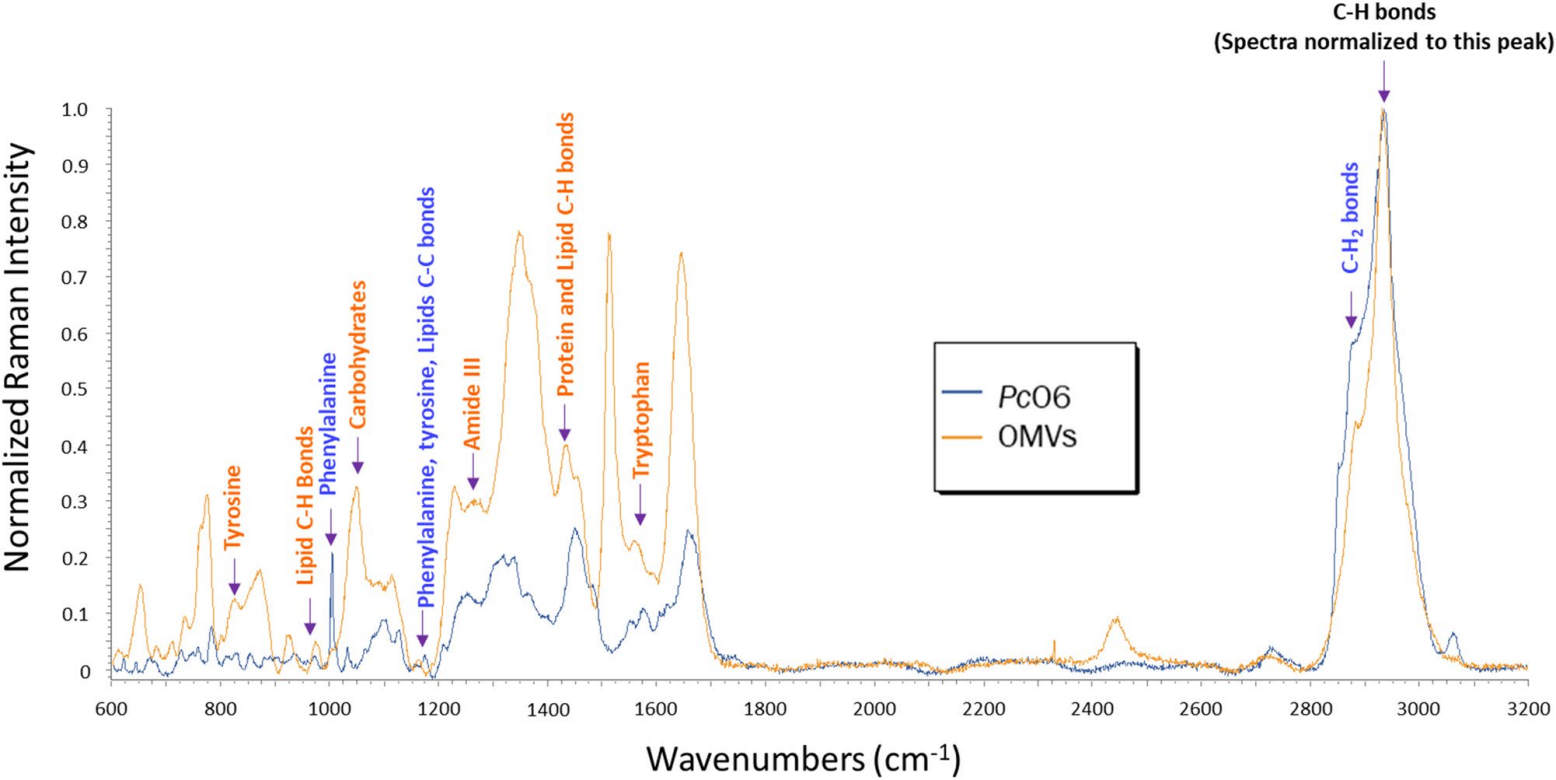
Comparisons between averaged Raman spectra of control *Pc*O6 cells and OMVs (n = 24 for *Pc*O6, n = 12 for OMVs). Peaks that are shared by *Pc*O6 and OMVs Raman spectra are marked with an arrow and labeled with the peak assignment. Text color indicates whether these peaks are higher in *Pc*O6 cells (blue text), or isolated OMVs (orange text). See Supplemental Table S1 for a list of all peak assignments and respective sources.

Raman spectroscopy was used to compare the chemical profiles of *Pc*O6 cells and isolated OMVs. Unique peaks were found in comparisons between Raman spectra of control biofilm *Pc*O6 cells and purified OMVs from control cells (Fig. 6): 27 peaks are unique to *Pc*O6 cells, 14 peaks are unique to OMVs, and 10 peaks are shared (a full list of peak assignments for *Pc*O6 cells and OMVs is shown in Supplemental Table S1). Figure 6 shows these shared peak assignments, and whether relative peak intensity is higher for control *Pc*O6 cells or resultant purified OMVs. The same trends were seen in H_2_O_2_ and CuO NP-treated *Pc*O6 cells and purified OMVs of stressed cells (data not shown).

**Figure 7:**
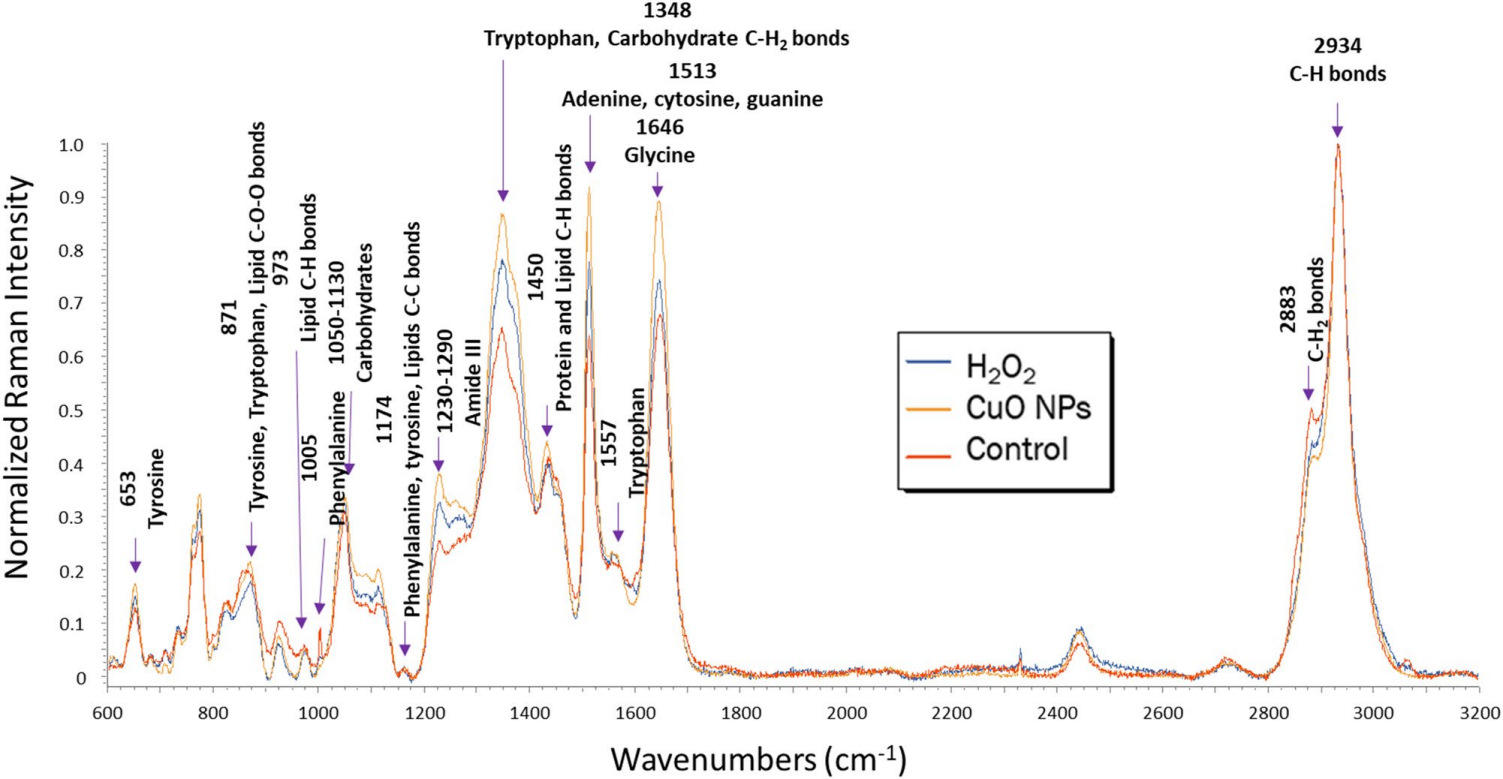
Averaged Raman spectra of OMVs harvested from *Pc*O6 colonies exposed to H_2_O_2_, CuO NPs, or no stressor at all (3 replicates with 4 spectra each for a total of n = 12) from 600 to 3200 cm^-1^. Differences are seen in normalized peak intensities indicating large differences in relative concentrations of compounds in the OMVs. Notable peak assignments are marked with an arrow and labeled with their respective peak number and peak assignment. For a list of all peak assignments and their sources, see Supplemental Table S1.

The relative intensity differed between shared peaks. All but two of these shared peaks had higher relative intensity for the OMV spectra than spectra of intact *Pc*O6 cells. Though there are few peaks shared by intact *Pc*O6 cells and purified OMVs, several unique peaks for *Pc*O6 and OMV Raman spectra correspond to the same chemical compounds: i.e. *Pc*O6 peak 644 cm^−1^ and OMV peak 870 cm^−1^ both correspond with tyrosine. Raman peaks assigned to the amino acid tryptophan and the nucleic acid bases adenine, cytosine, and guanine are present in spectra *Pc*O6 cells and purified OMVs. However, the peaks assigned to these chemistries differ between the spectra for intact biofilm cells and the isolated OMVs. Both *Pc*O6 and OMV spectra show various lipid, carbohydrate, protein, and nucleic acid signatures although different signatures of these macromolecules appear in each: i.e. *Pc*O6 peak 810 cm^−1^ indicates lipid O–P–O bonds while OMV peak 1092 cm^−1^ indicates lipid C–C bond (Supplemental Table S1).

Especially notable in Raman spectra of isolated OMVs was the increased intensity of nucleic acid peaks which implies that DNA and/or RNA are selectively packaged by *Pc*O6 into and/or onto OMVs. This conclusion was confirmed by measuring OMV 260/280 UV-absorption ratios, which compares the concentration of nucleic acids to the concentration of protein at their respective absorption peaks of 260 and 280 nm. OMV 260/280 ratios did not vary according to cell stressor (Supplemental Table S2) with an overall mean of 5.91 ± 0.09. Generally, a 260/280 ratio above 1.8 is obtained with a pure DNA sample and 2.0 is obtained with a pure RNA sample. The micro-BCA assay results confirmed varying amounts of protein in OMV suspensions (Supplemental Fig. S5b). These high 260/280 ratios for OMVs could be due to other compounds within OMVs that absorb light at similar wavelengths. *Pc*O6 is known to secrete several secondary metabolites including phenazines and siderophores^42^ and these results suggest that some metabolites may be packaged into OMVs.

### Chemical characterization of stress-induced outer membrane vesicles with Raman spectroscopy

The chemical profiles of control OMVs were compared with H_2_O_2_- and CuO NP-induced OMVs with Raman spectroscopy and LDA. Raman spectra of OMVs purified from control and stressed cells showed consistency similar to Raman spectra of control and stressed intact *Pc*O6 cells. Averaged Raman spectra (n = 12 from 3 replicates) from OMVs harvested from cells of all treatments are shown in Fig. 7 differences are seen in the peak intensity of control OMVs and those harvested from *Pc*O6 exposed to CuO NPs and H_2_O_2_, most notably: (a) 1646 cm^−1^, glycine; (b) 1513 cm^−1^, adenine, cytosine, and guanine; and (c) 1348 cm^−1^, adenine, guanine, tyrosine, and tryptophan. For these peaks, spectra of OMVs from CuO NP-treated *Pc*O6 had the highest intensity, followed by OMVs from H_2_O_2_-treated *Pc*O6 then OMVs of control cells.

**Figure 8:**
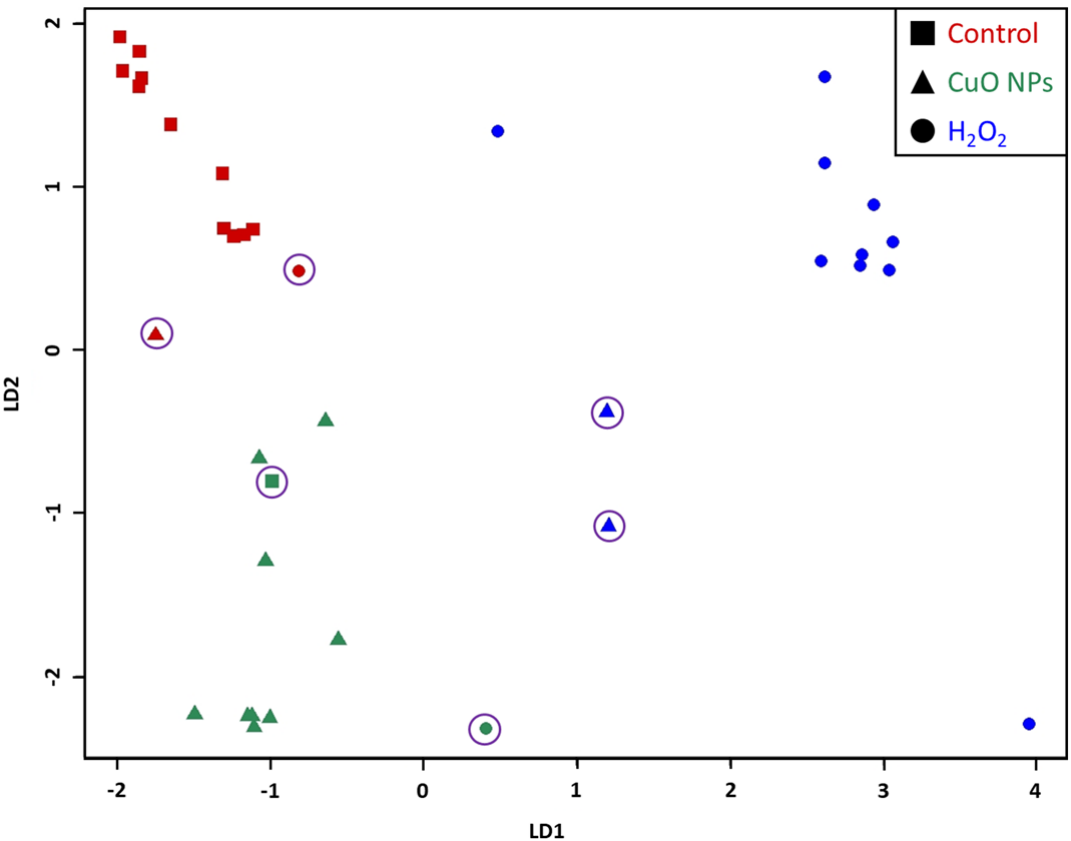
LDA plot of Raman spectra from purified OMVs (n = 12 for control and CuO NP-induced OMVs and n = 11 for H_2_O_2_-induced OMVs from three OMV isolations). Data were truncated to 750–1700 cm^−1^ and 2670–3100 cm^−1^, where most peaks are located. The shape of the points of the plot indicate the true treatment of each spectrum whereas the color of the point indicates the predicted treatment according to LDA. Misclassified spectra are circled in purple.

LDA results of the broad portion of the spectra (750–1700 & 2670–3100 cm^−1^) showed a 83.3% accuracy for pure OMVs from all treatments with several misclassified spectra in each treatment (LDA plot shown in Fig. 8, LDA confusion matrix shown in Table 2). Narrower portions of the spectra were also examined with LDA (Supplemental Fig. S6). Much like the LDA examination of *Pc*O6 spectra, LDA of different portions of the OMV spectra showed differences in the grouping on LDA plots. However, the accuracy of the algorithms did not vary greatly between the spectra portions (83–89% accuracy). Five of the six spectra that were misclassified in LDA of the broader spectra were misclassified in most or all of the narrower portions. The LDA plots of these narrower portions control spectra were far removed from the spectra of the other treatments. In contrast, the spectra of OMVs harvested from H_2_O_2_-treated and CuO NP-treated cells tended to be grouped apart as well though with a large intermingled section between them that contained many of the misclassified spectra.

**Table 2:** Confusion matrix from LDA plot of Raman spectra given by pure OMVs.

Confusion matrix of Raman spectra of pure OMVs				
		Predicted treatment		
		Control	CuO NPs	H_2_O_2_
True treatment	Control	11	1	0
	CuO NPs	1	9	2
	H_2_O_2_	1	1	10

Data were truncated to 750–1700 cm^−1^ and 2670–3100 cm^−1^, where most peaks are located. LDA of the Raman spectra gave an 83.3% accuracy indicating that spectra of OMVs are highly dependent on the cellular stressor.

Biochemical assays were also used to measure the LPS and protein content of OMVs. LPS content (average of 15.6 ± 2.7 endotoxin units (EU)/mL solution), which should correlate with OMV number, increased slightly in H_2_O_2_-induced OMVs (Supplemental Fig. S5a). This result matches literature sources that OMV production is increased in response to stress, including ROS stress^21,27,28^. Protein content (average of 23.3 ± 13.4 μg/mL solution) of OMVs was uncorrelated with cellular stressor and trial (Supplemental Fig. S5b) even when normalized to the LPS content (Supplemental Fig. S5c).

## Discussion

DLS and AFM measurements showed that OMV sizes were highly polydisperse in size regardless of the cellular stressor. Differing OMV diameter according to the analysis method has been reported in the literature^67^, likely due to differences in OMV aggregation and/or hydration. Aggregation of OMVs observed in AFM and SEM images may be due to outer membrane surface features similar to those involved in adhesion for intact cells^69^. Extracellular biomolecules within the biofilm matrix, which include proteins, polysaccharides, and eDNA^70^, may also bind to OMVs and cause this aggregation. Similar OMV aggregation was observed in studies of OMVs produced by *Neisseria lactamica* by Gorringe et al.^71^. The researchers observed that numerous large OMV aggregates occurred at pH 7.0 with fewer aggregates occurring at pH 8.0, implying that many OMV surface proteins reached an isoelectric equilibrium at this higher pH, thus reducing the number of OMV aggregates. LPS charge may also be altered by environmental pH, influencing electrostatic and steric barriers to agglomeration.

OMV aggregation may be counterproductive to signaling and similar OMV roles, however, OMV assembly may contribute to the assembly of scaffolds to create and support the biofilm matrix. OMVs produced by biofilm *Myxococcus xanthus* cells form chains that tether biofilm cells together^72^. *Xyllela fastidiosa* uses OMV aggregates to mediate surface adhesion, either promoting or preventing biofilm formation by the bacterium^17^. *Pc*O6 is a strong biofilm former^41^ and OMV release by this bacterium may promote its aggressive colonization of plant roots during adhesion and biofilm formation as patches on the plant root^40^.

This work established that Raman spectroscopy coupled with LDA was a reliable method for characterization of OMV content; the creation of Raman peak libraries (such as Supplemental Table S1 and references^73–76^) is valuable in assigning structural changes to cells under different exposures. In this study, LDA was particularly important as Raman spectra of stressed *Pc*O6 cells did not have different peaks from those of control cells, only peak intensities differed. This same pattern occurred for OMVs isolated from stressed and control cells.

To examine what Raman bands were most heavily weighted during LDA grouping calculations, LDA was also performed on smaller spectral regions (Supplemental Fig. S4). For the spectra of *Pc*O6 cells, it appears that LDA is primarily grouped based on peak differences in the range of 750–1700 cm^−1^. The 1400–1500 cm^−1^ and 1500–1700 cm^−1^ regions were especially unique for H_2_O_2_ and CuO NP treatments, respectively. LDA of these regions yielded distinct groupings with few miscalculations. For the OMV spectra, the LDA algorithm primarily converged on the 750–1700 cm^−1^ range as well. However, LDA of narrower spectral portions within this range did not improve LDA results, indicating that LDA groupings of OMV spectra are less reliant on single peaks and regions than LDA groupings of *Pc*O6 cells.

Comparing Raman spectra of intact *Pc*O6 cells and isolated OMVs, it is clear that certain *Pc*O6 cell components are enriched while others are excluded in OMVs, which agrees with studies of other Gram-negative bacteria^8,77^. The decreased peak intensity of H_2_O_2_-treated *Pc*O6 cells is likely because unsaturated lipids react with H_2_O_2_ to form lipid peroxides and reactive aldehydes^78^. The fact that the same peaks are affected in CuO NP-stressed *Pc*O6 cells likely indicates that ROS are formed in cells as a response to CuO NP stress, a cell response that contributes to the dose-dependent bactericidal activity of CuO NPs^79^. The relative greater content of unsaturated lipids in OMVs harvested from these stressed cells compared to OMVs produced by control cells could relate to changes in membrane integrity of cells under stress.

Also notable is the high amount of nucleic acids in OMVs compared to whole biofilm *Pc*O6 cells, implicating the potential role of OMVs to transport DNA and/or RNA in *Pc*O6 biofilms. RNA in OMVs has been reported in the forms of ribosomal RNA, mRNA, and small RNAs^80^. OMVs released by *P. aeruginosa* may contain plasmid DNA^12^, coding chromosomal DNA^5^, or noncoding eDNA^37^. These nucleic acids extend proposed OMV roles in cell-to-cell communication to both gene and transcription levels assuming OMV contents are taken up by live cells. OMVs of *P. aeruginosa* contain chromosomal DNA encoding genes related to bacterial survival under stress conditions^5^. The eDNA is an important part of the biofilm matrix^45^ and is involved in adhesion to surfaces, aggregation of bacterial cells, and the exchange of genetic information^70^. In *H. pylori*, eDNA was observed on OMV surfaces, with suggested roles in OMV aggregation and cell-to-cell binding^6^ including within the biofilm matrix^72^.

The biochemical assays confirmed the presence of protein, LPS, and nucleic acids in the OMVs from *Pc*O6 that are indicated from the Raman spectral peaks. The inability of these assays to discriminate among OMVs according to the cellular stressor is unsurprising: OMVs are chemically heterogeneous in nature^6,16,68^ due to the multiple potential mechanisms and pathways that lead to OMV formation^11,81,82^. The presence of LPS, lipoproteins, and DNA in OMVs is significant because these are among the structures known as MAMPs and DAMPs that induce plant innate resistance^35^. Thus, the contents of the OMVs released from *Pc*O6 cells may be implicated in the induction of systemic resistance observed in plants with roots colonized with *Pc*O6.

The findings of OMV production from the plant-beneficial microbe, *Pc*O6, and the changes in their composition with NP and ROS exposure revealed features that influence plant and bacterial responses in the rhizosphere. The nature of the charge on the OMV surfaces and the eDNA content might affect biofilm formation. Proteins such as catalase found in OMVs from other bacteria^28^ potentially protect the bacterial cell from oxidative damage. DNA and RNA release through OMVs could affect plant gene expression. These bacterial nucleic acids, along with other confirmed MAMPs such as LPS, carried by OMVs, could induce plant resilience to stress.

In summary, research into the heterogeneous nature and multiple roles of OMVs is supported by applying sensitive techniques such as Raman spectroscopy supported by appropriate analyzation algorithms such as LDA. In this study, these methods were able to chemically characterize purified OMVs and categorize these vesicles based on two cellular stressors relevant to the soil environment of the plant health-promoting bacterium, *Pc*O6. The ability to differentiate H_2_O_2_-induced and CuO NP-induced OMVs reveals that these stressors do not merely induce OMV production. Rather, *Pc*O6 responds to these stressors with changes in OMV contents and composition. The construction of an OMV Raman peak assignment library presented here provides a baseline for future analyses by our group and others.

## Methods

### Bacterial growth conditions

*Pc*O6 stocks were kept at − 80 °C in 15% glycerol and thawed before use. *Pc*O6 biofilms were grown at 22 °C on minimal medium (K_2_HPO_4_—10.5 g/L, KH_2_PO_4_—4.5 g, Na*citrate*2H_2_O—0.5 g/L, (NH_4_)_2_SO_4_—1 g/L, sucrose—2 g/L, anhydrous MgSO_4_—0.125 g/L) 2% agar plates (15 × 100 mm) for 48 h to a confluent lawn.

*Pc*O6 biofilms were also grown on hollow fiber membranes for SEM imaging^41^. Hollow fiber membranes were inoculated with 2 μL *Pc*O6 suspended in sterile double distilled H_2_O (ddH_2_O) (resistance > 18 MΩ cm) at a concentration of 10^6^ colony forming units (CFUs)/mL, draped across wells with liquid minimal medium, and allowed to grow at 22 °C for 28 h.

### Abiotic stressor preparations

Sterile ddH_2_O was used as the control treatment. Commercial CuO NPs (nominal size < 100 nm, 99.95% purity) were obtained from American Elements as a nanopowder and stored protected from light. NP size distribution and agglomeration profiles were confirmed with scanning electron microscopy (FEI Quanta FEG 650) (Supplemental Fig. S7). NP elemental composition was determined by scanning electron microscopy with energy-dispersive X-ray spectroscopy using an X-Max Detector (Oxford Instruments). NP stress used CuO NPs suspended in sterile ddH_2_O (30 mg Cu from CuO NPs/L) through sonication (Q500, QSonica LLC) for 10 min with an alternating 10 s on/off cycle at 25% amplitude. The ROS stress was 3% H_2_O_2_ (v/v).

### Treatments of *Pc*O6 biofilm cells before OMV isolation

The confluent lawns of *Pc*O6 biofilm cells on minimal medium agar were treated by flooding with 10 mL of ddH_2_O, H_2_O_2_, or CuO NPs, added in 1 mL aliquots. These conditions were maintained for 60 min, during which time the cells detached from the surface and became suspended in the liquid. CFUs were counted with serial dilutions on LB 2% agar petri dishes to check the effect of stressors on cell viability. CuO NPs had no significant effect on the ability of *Pc*O6 to generate colonies on plate medium whereas H_2_O_2_ exposure reduced colonies < 10%.

### Isolating and purifying outer membrane vesicles

OMVs were isolated from *Pc*O6 cells using a method adapted from Zhou et al.^83^. Twenty minimal medium plates with confluent *Pc*O6 lawns (a total surface area of 0.628 m^2^) were used per treatment to maximize OMV yields for analysis. The cell suspension (10 mL/plate for a total of 200 mL/treatment) was poured from the petri dishes into 50 mL polypropylene conical tubes and centrifuged (10,000×*g*, 20 min) to generate a cell pellet leaving OMVs and other secreted materials in the supernatant. This step also removed CuO NPs from the solution when this stressor was used. The OMVs were concentrated from the supernatant by ammonium sulfate precipitation. Ammonium sulfate (Mallinckrodt chemicals) was added over the course of two hours (240 g/L total) at 15 min intervals to the supernatant, which was left undisturbed at 22 °C until precipitates formed (anywhere from 2–8 h). The precipitates were pelleted by centrifugation (10,000×*g*, 10 min), resuspended in 1 mL sterile deionized H_2_O, and dialyzed against ddH_2_O for at least 16 h. The solution was sterile filtered (0.45 μm, Ultrafree PVDF centrifugal filter units, Beckman Coulter Inc.) to remove any remaining cells or contaminants.

To purify OMVs from flagella, pili, and secondary metabolites (Supplemental Fig. S8), density gradient ultracentrifugation was performed using methods adapted from Chutkan et al.^84^. The crude OMV suspension was mixed with OptiPrep iodixanol gradient medium (Sigma Aldrich) to create a 45% OptiPrep solution (vol/vol). A 2 mL aliquot was loaded into the base of each ultracentrifuge tube (Ultraclear 12.5 mL centrifuge tube, Beckman Coulter) and covered sequentially with 2 mL layers of 40, 35, 30, 25, and 20% OptiPrep before centrifugation (212,000×*g*, 3 h, 4 °C) (Optima LE-80 K Centrifuge, Beckman). Aliquots of 1 mL were collected from the top of the gradient in a cold room to minimize diffusion. OMV-containing fractions, the top 1 mL of each tube, were pooled from each treatment, diluted at least 10 times with sterile deionized H_2_O, loaded into centrifuge tubes (26.3 mL Polycarbonate Bottle with Cap, Beckman Coulter), and centrifuged (40,000×*g*, 3 h) to pellet the OMVs. The OMV pellet was resuspended in 750 μL sterile ddH_2_O and sterile filtered (0.45 μm, Ultrafree PVDF centrifugal filter units). The presence of OMVs in the filtrate was confirmed with AFM imaging. Pure OMV preps were stored at 4 °C until used for Raman spectroscopy, which took place within 48 h of OMV isolation and purification. Samples were frozen (− 20 °C) until used for biochemical assays.

### Atomic force microscopy (AFM)

AFM was performed on a Nanoscope III Bioscope (Digital Instruments, Inc.) in tapping mode. Budget Sensors-Tap 300AL-G cantilevers with a tip radius of curvature < 10 nm, length of 125 μm, width of 30 μm, thickness of 4 μm, and a 40 N/m force constant were employed. Images were collected at 256 × 256 resolution and 1 Hz over a range of scan sizes and scan angles. For intact biofilm *Pc*O6 cells, a sample of the bacterial lawn on a sterile inoculation loop was smeared on a clean glass slide and immediately imaged. For OMVs, 20 μL purified OMV suspension was pipetted onto a clean glass slide, allowed to dry, and immediately imaged. OMV diameter was determined using the horizontal distance and the vertical distance in the line cut feature in the Nanoscope software (Supplemental Fig. S9). Four images were used for each treatment with a total of 100 OMVs measured per treatment.

### Scanning electron microscopy (SEM)

SEM was performed with an FEI Quanta FEG 650 equipped with an Oxford X-Max EDS housed in the Microscopy Core Facility at Utah State University. Hollow fiber membranes with *Pc*O6 biofilms were dipped in sterile distilled H_2_O, fixed in methanol for 10 min, and dried in two 10 min bath of 100% ethanol. Samples were critical point dried and sputter coated in an Au/Pd coat. Images were captured under high vacuum. Purified OMVs were pipetted onto a clean substrate, fixed with 2.5% glutaraldehyde, dehydrated using two 5 min washes of increasing ethanol concentrations (50, 70, & 95%) followed by three 15 min washes with 100% ethanol. Samples were imaged under high vacuum without coatings.

### Dynamic light scattering

DLS measurements were performed on a DynaPro NanoStar (Wyatt Technology Corporation, Santa Barbara, CA) employing Dynamics Software (version 7.0.3, Wyatt Technology Corporation, Santa Barbara, CA) and a 658 nm laser. The purified OMV suspensions were diluted 1:10 in sterile deionized water, and 70 μL was transferred to DLS cuvettes. The intensity autocorrelation function (see Supplemental Fig. S10 for autocorrelation graphs) was used to calculate a hydrodynamic diameter based on the Stokes–Einstein equation using a regularization method employed in the software.

### Raman spectroscopy and linear discriminant analysis (LDA)

To examine the intact cells, confluent *Pc*O6 lawns from two minimal medium plates with or without the stress treatments were centrifuged from solution (14,000×*g*, 10 min), and resuspended in 1 mL sterile, deionized H_2_O. This process was repeated two additional times to remove stressors. After the third centrifugation, *Pc*O6 cells were resuspended in 200 μL sterile, deionized H_2_O. A total of 6 *Pc*O6 replicates were performed per treatment. The majority of OMVs, cell secretions, and other cell debris remained in the supernatant which was discarded after each centrifugation. A 10 μL aliquot of the *Pc*O6 suspension was pipetted onto aluminum tape affixed to a clean glass microscope slide and allowed to dry. For OMVs, no additional preparation was needed for Raman spectroscopy after isolation and purification steps. A total of 3 isolations were performed with each treatment. Twenty μL purified OMV suspension was pipetted onto aluminum tape affixed to a clean glass microscope slide and allowed to dry.

Raman spectra were obtained using a Renishaw inVia Raman microscope with a 633 nm laser, 1200 g/mm grating, and 14 mW laser power. Spectra were obtained over a 30 s acquisition time with a wavenumber range of 200–3200 cm^−1^, with 3 accumulations per spectrum. Four spectra were captured per treatment during each replicate for a total of n = 24 for *Pc*O6 spectra and n = 12 for OMV spectra. Spectrogryph^85^ was used to visualize spectra, obtain peak numbers, and average spectra. LDA of spectra was performed with R following background subtraction (WiRE 4.1), removal of cosmic rays (WiRE 4.1), and normalization of spectra (R). Data were truncated to 750–1700 cm^−1^ and 2670–3100 cm^−1^ for broad-spectrum analysis. *Pc*O6 cell spectra were further truncated to 750–1700 cm^−1^, 2670–3100 cm^−1^, 700–950 cm^−1^, 950–1200 cm^−1^, 1200–1400 cm^−1^, 1400–1500 cm^−1^, or 1500–1700 cm^−1^ for additional examination with LDA. OMV spectra were further truncated to 750–1700 cm^−1^, 2670–3100 cm^−1^, 1000–1200 cm^−1^, 1200–1500 cm^−1^, 1500–1600 cm^−1^, or 1500–1600 cm^−1^ for additional examination with LDA.

### Micro-bicinchoninic acid (micro-BCA) assay

OMV protein content was quantified with a Pierce micro-BCA assay kit (Thermo Scientific). Frozen OMV samples were thawed and prepared according to the manufacturer's instructions and absorbance was read at 562 nm (Synergy HT, BioTek Instruments Inc.) with appropriate bovine serum albumin standards from the manufacturer with concentrations of 0 to 200 μg protein/mL. Duplicates were run for each standard and sample.

### Lipopolysaccharide (LPS) quantification

OMV LPS content was quantified with a Pierce Endotoxin Quantification Kit (Thermo Scientific). Frozen samples were thawed and prepared according to the manufacturer's instructions and absorbance was read at 405 nm (Synergy HT, BioTek Instruments Inc.) with LPS standards from *Escherichia coli* supplied by the manufacturer in the range of 0.1 to 1 Endotoxin Units (EU)/mL. Duplicates were run for each standard and sample.

### 260/280 absorption ratio

The nucleic acid to protein ratio was determined based on the 260/280 UV-absorption ratio. Frozen samples were thawed and 2 μL of each sample was pipetted into wells of a Take3 Micro-Volume plate (BioTek Instruments Inc.) and absorbance was read at 260 and 280 nm (Synergy HT, BioTek Instruments Inc.). Standards of bovine serum albumin (40 μg/mL) from a micro-BCA assay kit (Thermo Scientific) and double-stranded DNA (100 μg/mL) from a PicoGreen DNA quantification kit (Thermo Scientific) were also run. Duplicates were run for each sample and standard.

## Supplementary Information

Supplementary Information.

## Data Availability

The datasets generated during this study are available from the corresponding authors on appropriate request.
