# Supplementary material for: Abiotic stressors impact outer membrane vesicle composition in a beneficial rhizobacterium: Raman spectroscopy characterization
Source: Sci Rep. 2020 Dec 4;10:21289. doi: 10.1038/s41598-020-78357-4 (PMC7719170; doi:10.1038/s41598-020-78357-4)
Supplement: Supplementary file 1 — Supplementary Information. [file 41598_2020_78357_MOESM1_ESM.pdf]

Abiotic stressors impact outer membrane vesicle composition in a beneficial rhizobacterium: Raman spectroscopy characterization

Matthew Potter[1], Cynthia Hanson[1], Anne J. Anderson[1], Elizabeth Vargis[1]*, David W. Britt[1]*

[1]Department of Biological Engineering, Utah State University, Logan, UT 84322, United States
*Co-corresponding authors

Contact information:

- Matthew Potter- m.k.p@aggiemail.usu.edu
- Cynthia Hanson- chanson8491@gmail.com
- Anne J. Anderson- annejanderson33@gmail.com
- Elizabeth Vargis- vargis@usu.edu
- David Britt- david.britt@usu.edu

Supplemental Figures and Tables

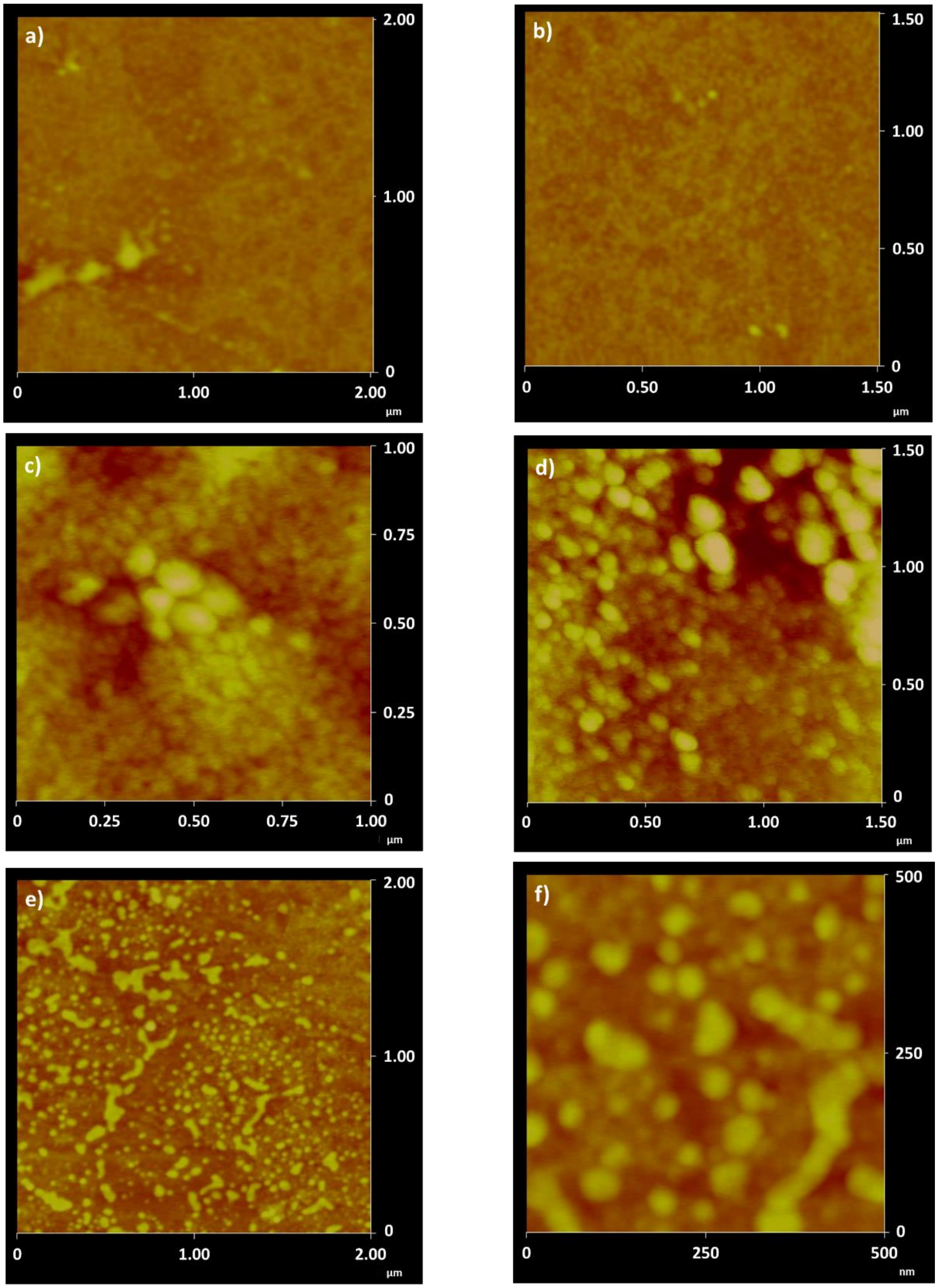

Supplemental Figure S1: AFM height images of purified OMVs. X- and y- scales are indicated in each image. Z-scales are from red to yellow from 0 to 25 nm respectively. a) & b) OMVs harvested from *Pc*O6 without any stressors. c) & d) OMVs harvested from *Pc*O6 under H_2O_2 stress (3% v/v). e) & f) OMVs harvested from *Pc*O6 under CuO NP stress (30 mg Cu/L).

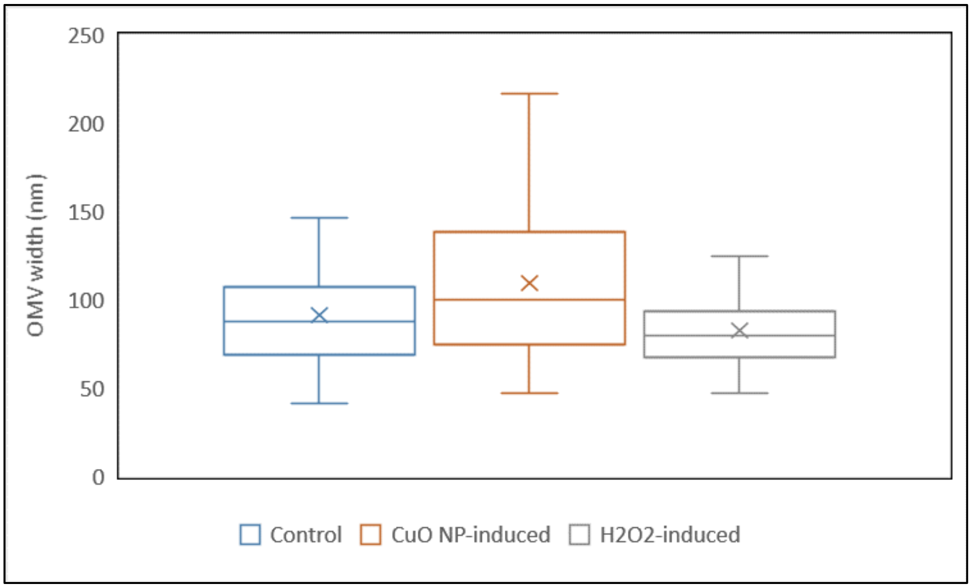

Supplemental Figure S2: Diameters of OMVs purified from *Pc*O6 cells subjected to the indicated stressors in AFM images. Diameters were measured by the AFM NanoScope software line cutting function (n=100 per treatment).

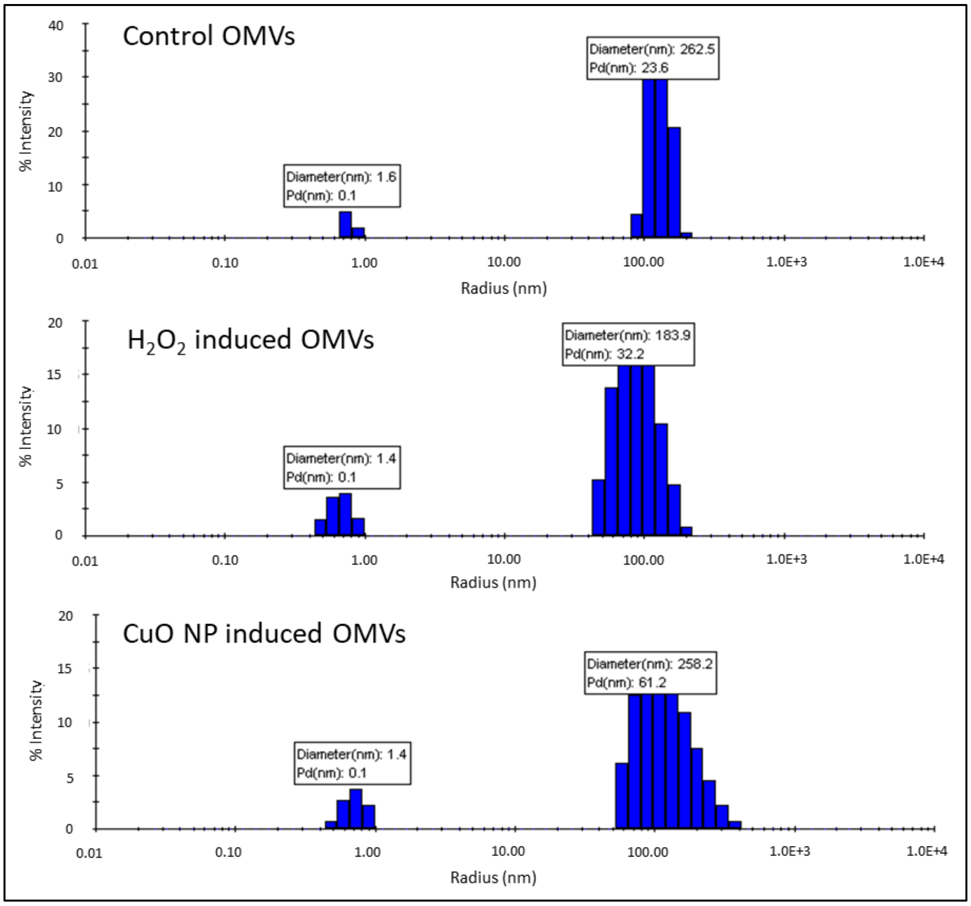

Supplemental Figure S3: Dynamic light scattering measurements of OMV diameters.

Supplemental Table S1: Peaks from Raman spectra of intact *Pc*O6 cells and purified OMVs of control cells with corresponding wave assignments from the literature. Other treatments are not shown because, as seen in **Error! Reference source not found.** and **Error! Reference source not found.**, all treatments have the same peaks though with differing intensities.

***Pc*O6 Peak (cm^{-1})**	**OMV Peak (cm^{-1})**	**Literature Peak (cm^{-1})**	**Assignment from Literature**	**Source**
623	-	623	Phenylalanine (C-C bond twist)	82
644	-	644, 645	Tyrosine (C-C bond twist)	82,83
-	653	654	Tyrosine	83
669	-	669	Thymine and guanine	82
-	685	683	Guanine (ring breath)	84
728	-	728	Adenine, and tryptophan (ring breath)	82
			Lipid C-N head groups	85
-	734	-	-	-
749	749	748	Tryptophan	83
758	-	758	Carbohydrate C-O ring (stretching)<br>Carbohydrate C-O-C, C-C-O, and O-C-O bonds (in-plane bending)	86
		759	Tryptophan	83
		760	Tyrosine (ring breath)	82
			Lipid $N^+(CH_3)_3$ bonds (symmetric stretching)	87
-	775	-	-	-
782	-	782	Uracil, cytosine, and thymine (ring breath)	82
810	-	811	Lipid O-P-O bonds	82
829	826	827	Tyrosine	83
		828	DNA O-P-O bonds (stretching)<br>Tyrosine (out of plane ring breath)	82
		829	Tyrosine	83
		830	Exposed tyrosine	88
853	-	853	Tyrosine	83
			Tyrosine (ring breath)	82
-	871	870	Tyrosine	83
		871	C-O-O lipid bonds (skeletal)	87
880	-	880	Tryptophan	83
		882	Lipid $N^+(CH_3)_3$ bonds (asymmetric stretching)	87
890-900	-	897	DNA deoxyribose phosphate backbone	82
			Proline	89
-	924	924	Protein N-C_α-C bonds	83
936	-	934	Protein C-C backbone	89
		936	Protein N-C_α-C bonds	83
		936	Carbohydrate C-O in-glycosidic linkage (stretching)	86
		937	Protein C-C α helix backbone (stretching)	82

Supplemental Table S1: Peaks from Raman spectra of intact *PcO6* cells and purified OMVs of control cells with corresponding wave assignments from the literature. Other treatments are not shown because, as seen in Error! Reference source not found. and Error! Reference source not found., all treatments have the same peaks though with differing intensities.

***Pc*O6 Peak (cm^{-1})**	**OMV Peak (cm^{-1})**	**Literature Peak (cm^{-1})**	**Assignment from Literature**	**Source**
973	974	974	Lipid C-H bonds (bending)	[87]
1004	1005	1004<br>1005	Phenylalanine	[82,83,88]
1050-1130	1030-1150	1030-1130	Carbohydrates	[90]
1064	-	1061	C-N and C-C bonds (stretching)	[88]
		1064	Lipid C-C bonds (stretching)	[87]
		1066	Protein C-N bond (stretching)<br>Lipid C-C chain bond (stretching)	[82]
-	1092	1095	DNA PO_2^- bond (stretching)<br>Lipid C-C chain bond (stretching)<br>Carbohydrate C-C bond (stretching)	[82]
		1090,1091,1094	Lipid C-C bond (stretching)	[87]
1100	-	1102	> PO_2^- (symmetrical stretching)	[88]
-	1120	1120	Lipid C-C bond (stretching)	[87]
1127	-	1127	Carbohydrate C-O bond (stretching)	[86]
		1127	Protein C-N bond	[83]
		1128	Protein C-N bond stretching<br>Lipid C-C chain bond stretching	[82]
		1130	Unsaturated fatty acid =C-C= bonds	[90]
1156	-	1145-1160	C-C and C-O bonds (ring breath, asymmetrical)	[91,92]
		1154	C-C, C-N, and $C-H_3$ bonds (stretching)	[88]
		1156	Protein C-C & C-N bonds (stretching)	[82]
		1155-1157	Sarcinaxanthin and carotenoids C-C bonds (stretching)	[92]
-	1162	1161	Sucrose fructosyl unit	[86]
1174	1172	1175	Tyrosine, phenylalanine	[85]
		1175	Lipid C-C bond (stretching)	[87]
		1176	Tyrosine C-H in plane bend	[82]
1209	-	1209	Phenylalanine and Tryptophan $C-C_6H_5$ bond (stretching)	[82]
		1211	Tyrosine	[83]
1220-1280	1230-1290	1220-1290	Amide III	[90]
1252	-	1252	Lipid =CH bond (deformation)	[87]
1330-1340	-	1336-1339	Adenine, guanine, tyrosine, tryptophan	[62,85]
-	1348	1348	Tryptophan C_α-H bond (deformation)	[83]
		1348	Carbohydrate $C-H_2$ bonds (wagging)	[86]
1365	-	1367	Lipid CH_3 bonds (symmetrical stretching)	[82]

Supplemental Table S1: Peaks from Raman spectra of intact *PcO6* cells and purified OMVs of control cells with corresponding wave assignments from the literature. Other treatments are not shown because, as seen in Error! Reference source not found. and Error! Reference source not found., all treatments have the same peaks though with differing intensities.

***Pc*O6 Peak (cm^{-1})**	**OMV Peak (cm^{-1})**	**Literature Peak (cm^{-1})**	**Assignment from Literature**	**Source**
1450	1430-1455	1431-1481	Protein marker band	[85]
		1449	Protein and lipid C-H bonds (deformation)	[82]
		1440-1460	C-H_2 bonds (deformation)	[88]
1482	-	1482	Protein C-H bonds (deformation)	[83]
-	1513	1518	Adenine, cytosine, guanine	[85]
1553	1557	1553, 1557	Tryptophan (indole ring)	[83]
1576	-	1575-1578	Guanine, and adenine (ring stretching)	[88]
		1578	Guanine, and adenine	[82]
1605	-	1605-1606	Phenylalanine	[88]
		1605	Tyrosine, tryptophan, and phenylalanine	[83]
1620	-	1617	Tyrosine and tryptophan C=C bonds	[82]
		1620	Tyrosine, tryptophan, phenylalanine	[83]
-	1646	1647	Glycine C=O bond	[93]
1660	-	1658	Unsaturated lipids	[94]
		1659	Amide I protein α helix	[82]
			Lipid C=C bonds (stretching)	
		1660	C=C lipid bond (stretching)	[87]
		1660	Protein amide I bond	[83]
		1650-1680	Amide I	[88]
		1663	Amide I	[94]
-	2446	-	-	-
2700-2770	2700-2790	-	-	-
2855	-	2855	Lipid C=H_2 bonds (asymmetric stretching)	[87]
2875	2883	2870-2890	C-H_2 bonds (stretching)	[88]
		2875, 2883	Lipid C=H_2 bonds (asymmetric stretching)	[87]
2935	2934	2935	C-H bonds (stretching)	[62,88]
		2935	Lipid C=H_3 bonds (symmetric stretching)	[87]
3062	-	3059	Aromatic C=C-H bonds (stretching)	[88]

Supplemental Figure S4: LDA plots and confusion matrices showing LDA results examining narrow portions of Raman spectra from whole *Pc*O6 cells. The examined wavenumbers are indicated for each set of results. Misclassified spectra are circled in LDA plots.

a)

750-1700 cm^{-1}

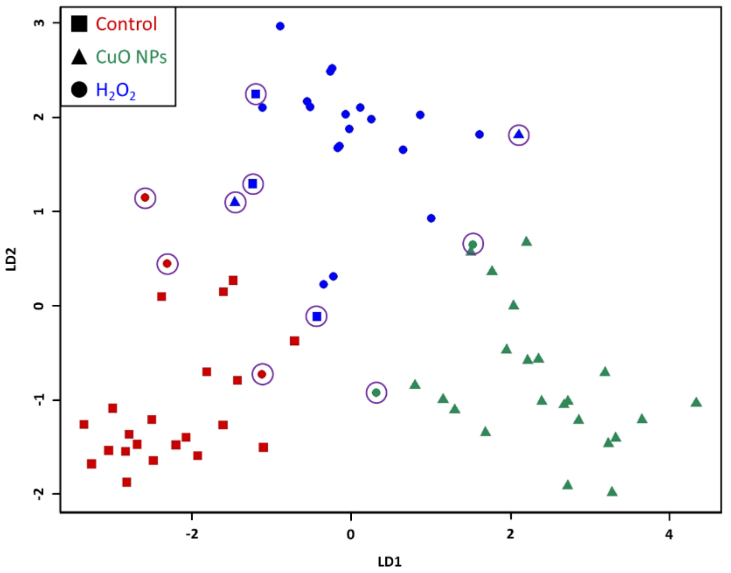

		Predicted Treatment		
		Control	CuO NPs	H_2O_2
True Treatment	Control	21	0	3
	CuO NPs	0	22	2
	H_2O_2	3	2	19

b)

2670-3100 cm^{-1}

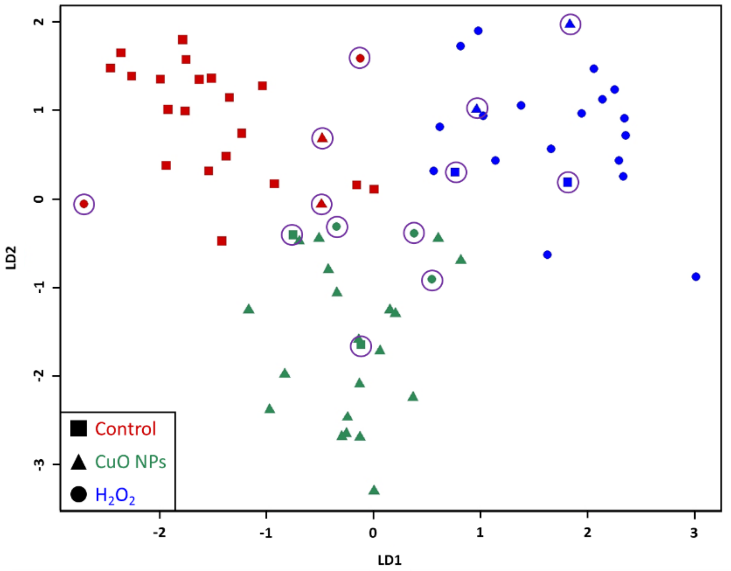

		Predicted Treatment		
		Control	CuO NPs	H_2O_2
True Treatment	Control	20	2	2
	CuO NPs	2	20	2
	H_2O_2	2	3	19

Supplemental Figure S4: LDA plots and confusion matrices showing LDA results examining narrow portions of Raman spectra from whole *PcO6* cells. The examined wavenumbers are indicated for each set of results. Misclassified spectra are circled in LDA plots.

c)

700-950 cm^{-1}

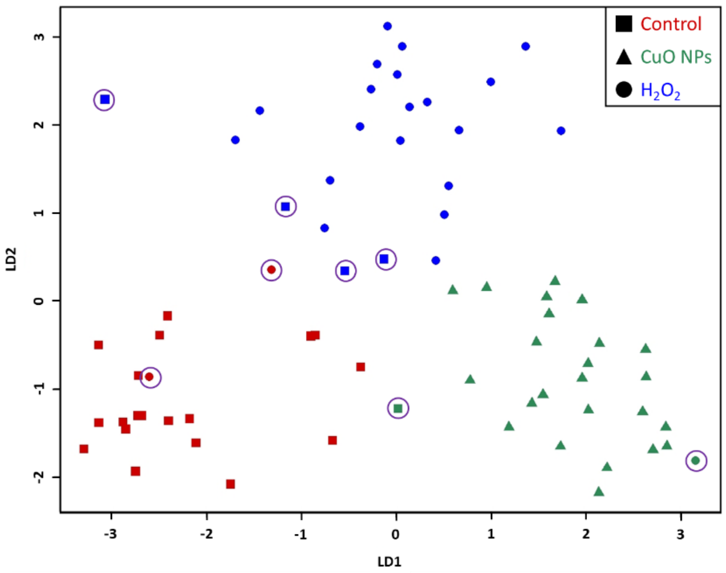

		Predicted Treatment		
		Control	CuO NPs	H_2O_2
True Treatment	Control	19	1	4
	CuO NPs	0	24	0
	H_2O_2	2	1	21

d)

950-1200 cm^{-1}

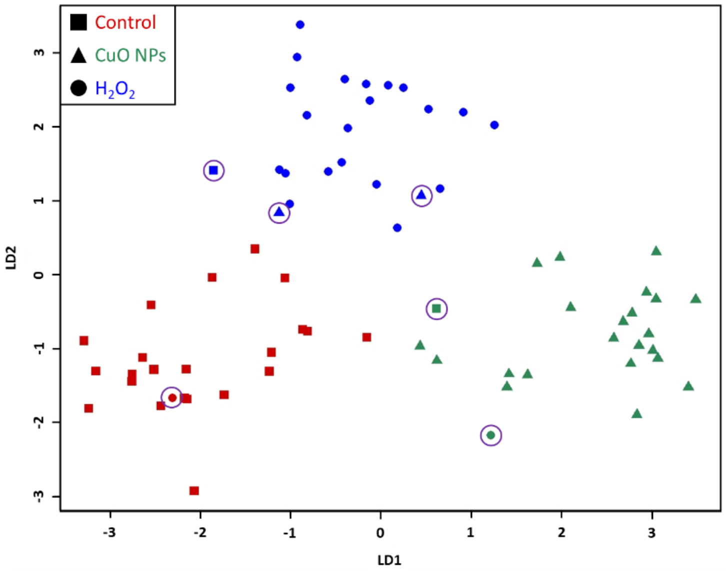

		Predicted Treatment		
		Control	CuO NPs	H_2O_2
True Treatment	Control	22	1	1
	CuO NPs	0	22	2
	H_2O_2	1	1	22

Supplemental Figure S4: LDA plots and confusion matrices showing LDA results examining narrow portions of Raman spectra from whole *PcO6* cells. The examined wavenumbers are indicated for each set of results. Misclassified spectra are circled in LDA plots.

e)

1200-1400 cm^{-1}

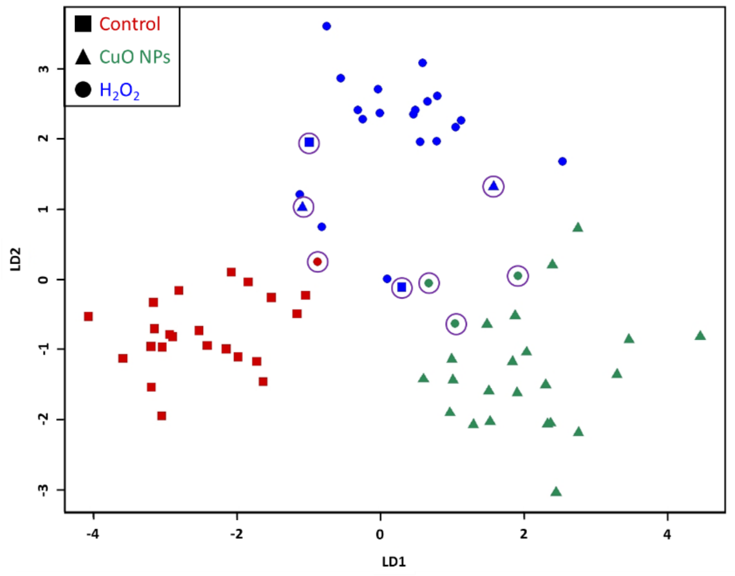

		Predicted Treatment		
		Control	CuO NPs	H_2O_2
True Treatment	Control	22	0	2
	CuO NPs	0	22	2
	H_2O_2	1	3	20

f)

1400-1500 cm^{-1}

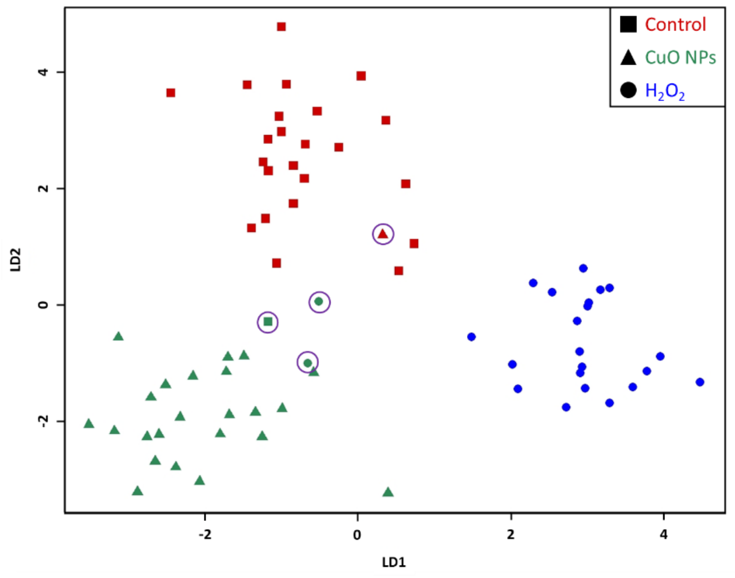

		Predicted Treatment		
		Control	CuO NPs	H_2O_2
True Treatment	Control	23	1	0
	CuO NPs	1	23	0
	H_2O_2	0	2	22

Supplemental Figure S4: LDA plots and confusion matrices showing LDA results examining narrow portions of Raman spectra from whole *PcO6* cells. The examined wavenumbers are indicated for each set of results. Misclassified spectra are circled in LDA plots.

g)

1500-1700 cm^{-1}

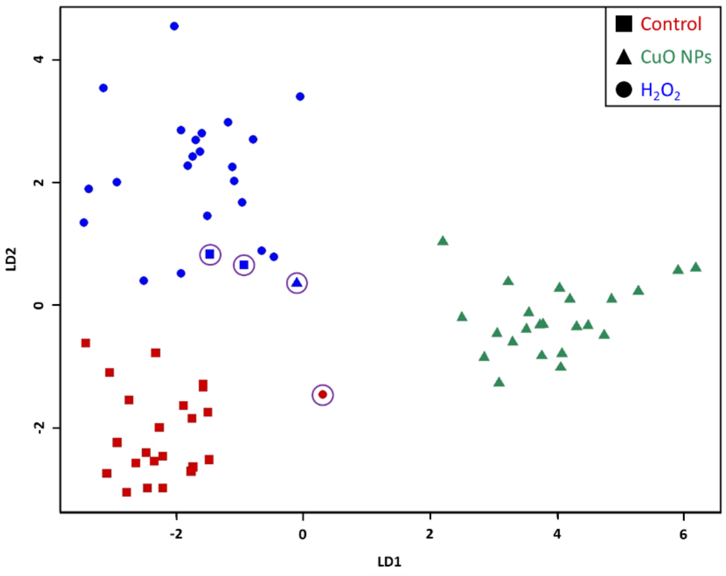

		Predicted Treatment		
		Control	CuO NPs	H_2O_2
True Treatment	Control	22	0	2
	CuO NPs	0	23	1
	H_2O_2	1	0	23

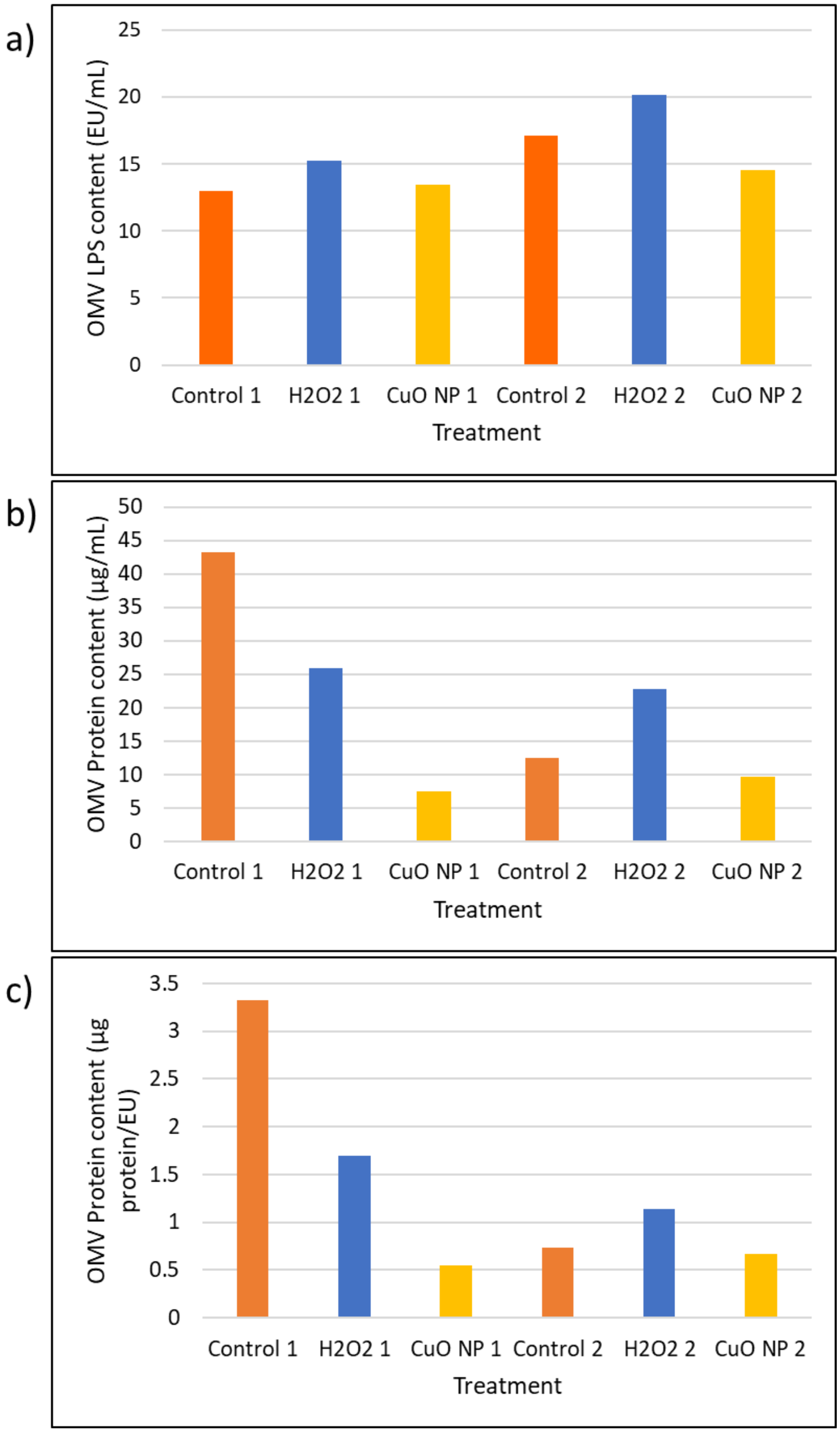

Supplemental Figure S5: a) OMV LPS content in endotoxin units (EU)/mL solution, b) OMV protein content in µg protein/mL solution, and c) OMV protein content normalized to LPS content, which should directly correlate with OMV number, in µg protein/EU. Results are from two separate OMV isolations.

Supplemental Table S2: 260/280 ratios of OMVs which quantify the nucleic acid to protein ratio respectively. Bovine serum albumin (BSA) and double stranded DNA (dsDNA) standards are also shown. Results are from two separate OMV isolations.

**Sample**	**260/280 ratio**
BSA standard (40 μg/mL)	1.443
	1.21
dsDNA sample (100 μg/mL)	2.043
	2.065
Control 1	5.886
	5.999
Control 2	5.92
	5.637
H_2O_2 1	5.987
	5.905
H_2O_2 2	5.945
	5.935
CuO NPs 1	5.969
	5.928
CuO NPs 2	5.884
	5.911

Supplemental Figure S6: LDA plots and confusion matrices showing LDA results examining narrow portions of Raman spectra from isolated OMVs. The examined wavenumbers are indicated for each set of results. Misclassified spectra are circled in LDA plots.

a)

750-1700 cm^{-1}

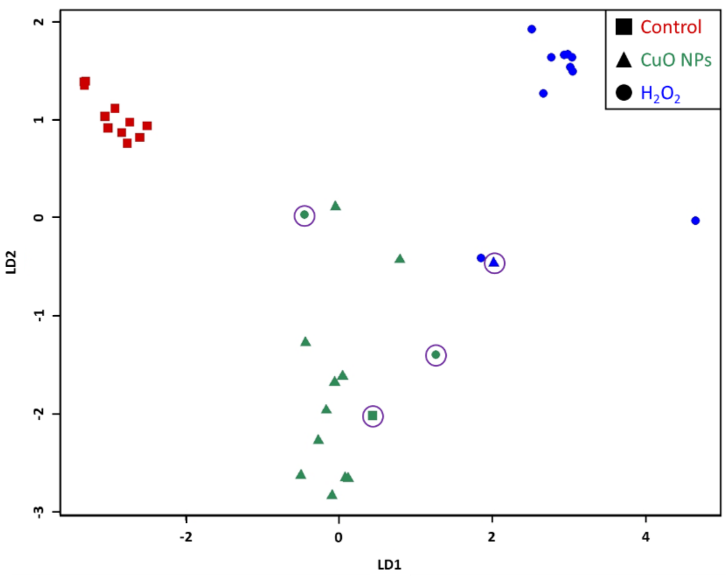

		Predicted Treatment		
		Control	CuO NPs	H_2O_2
True Treatment	Control	11	1	0
	CuO NPs	0	11	1
	H_2O_2	0	2	10

b)

2670-3100 cm^{-1}

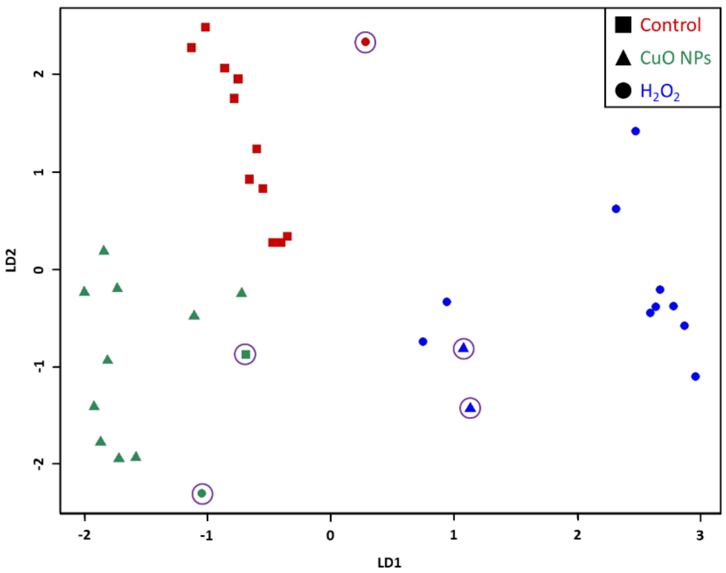

		Predicted Treatment		
		Control	CuO NPs	H_2O_2
True Treatment	Control	11	1	0
	CuO NPs	0	10	2
	H_2O_2	1	1	10

Supplemental Figure S6: LDA plots and confusion matrices showing LDA results examining narrow portions of Raman spectra from isolated OMVs. The examined wavenumbers are indicated for each set of results. Misclassified spectra are circled in LDA plots.

c)

1000-1200 cm^{-1}

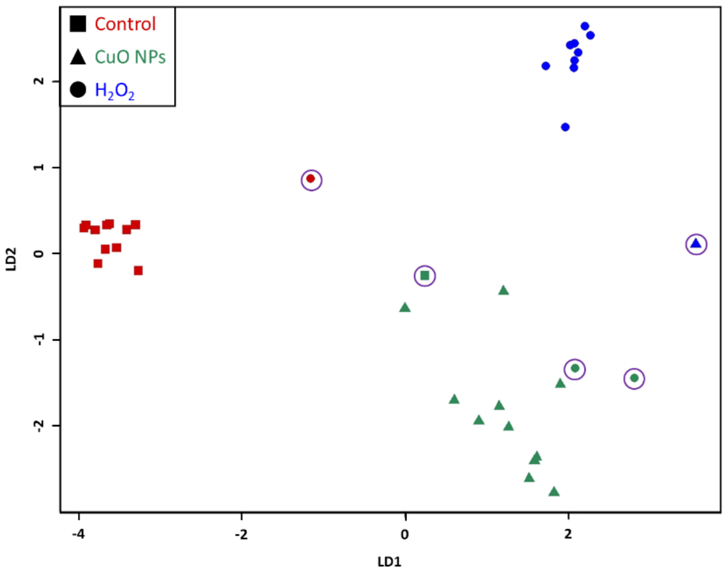

		Predicted Treatment		
		Control	CuO NPs	H_2O_2
True Treatment	Control	11	1	0
	CuO NPs	0	11	1
	H_2O_2	1	2	9

d)

1200-1500 cm^{-1}

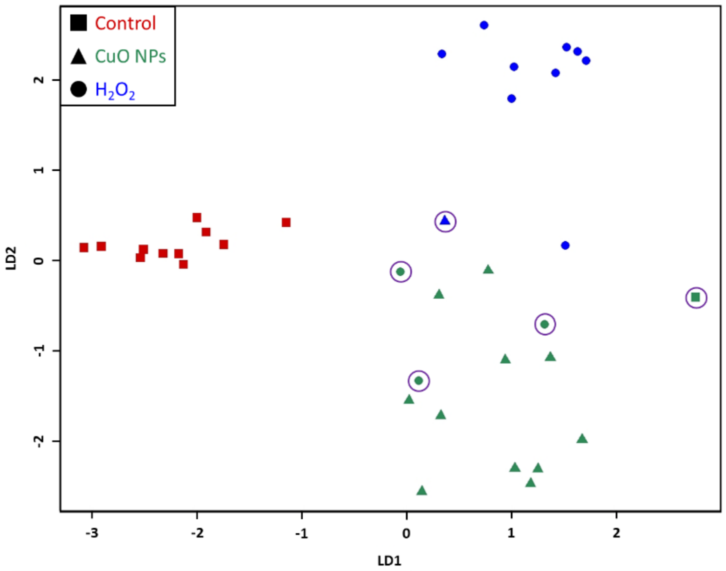

		Predicted Treatment		
		Control	CuO NPs	H_2O_2
True Treatment	Control	11	1	0
	CuO NPs	0	11	1
	H_2O_2	0	3	9

Supplemental Figure S6: LDA plots and confusion matrices showing LDA results examining narrow portions of Raman spectra from isolated OMVs. The examined wavenumbers are indicated for each set of results. Misclassified spectra are circled in LDA plots.

e)

1500-1600 cm^{-1}

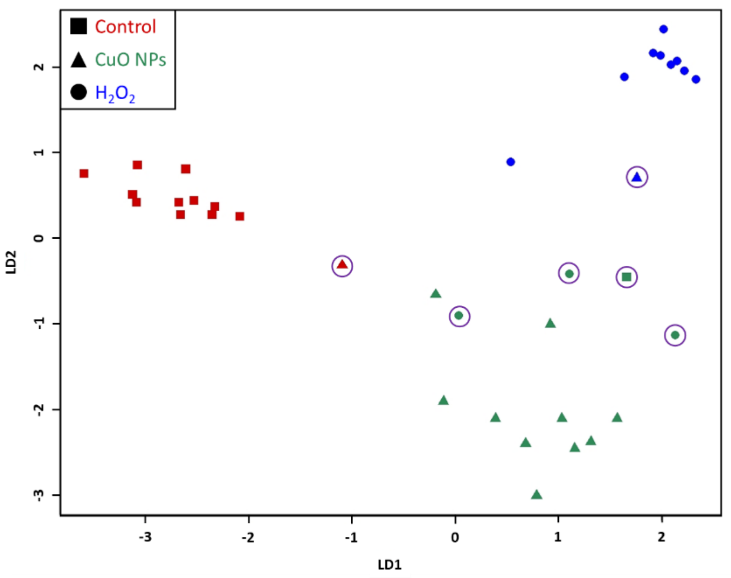

		Predicted Treatment		
		Control	CuO NPs	H_2O_2
True Treatment	Control	11	1	0
	CuO NPs	1	10	1
	H_2O_2	0	3	9

f)

1600-1700 cm^{-1}

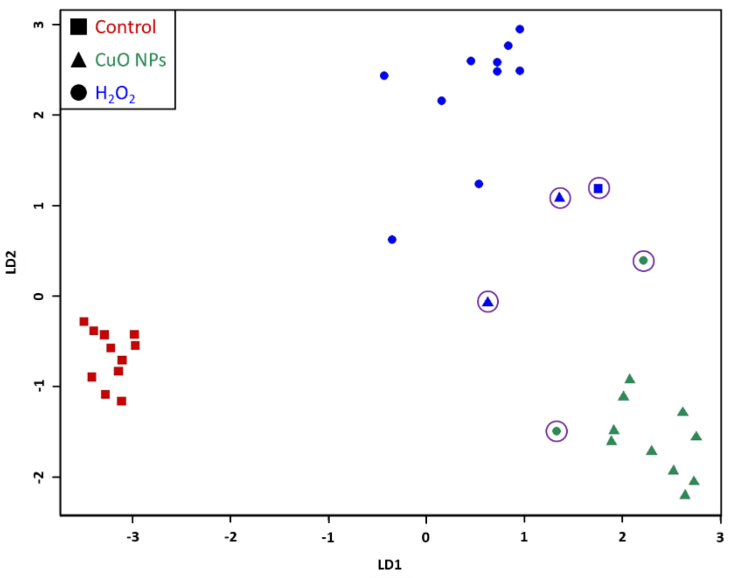

		Predicted Treatment		
		Control	CuO NPs	H_2O_2
True Treatment	Control	11	0	1
	CuO NPs	0	10	2
	H_2O_2	0	2	10

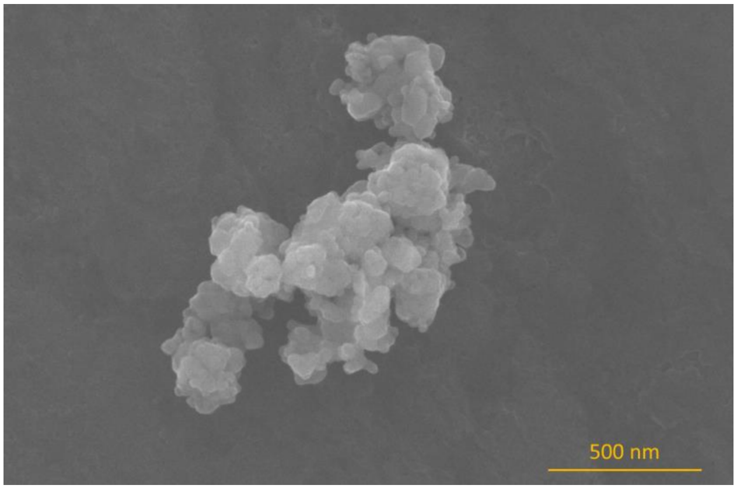

Supplemental Figure S7: SEM image of CuO NPs. The scale bar is shown in the lower right corner. This and other similar images were used to confirm the CuO NP size range given by the manufacturer.

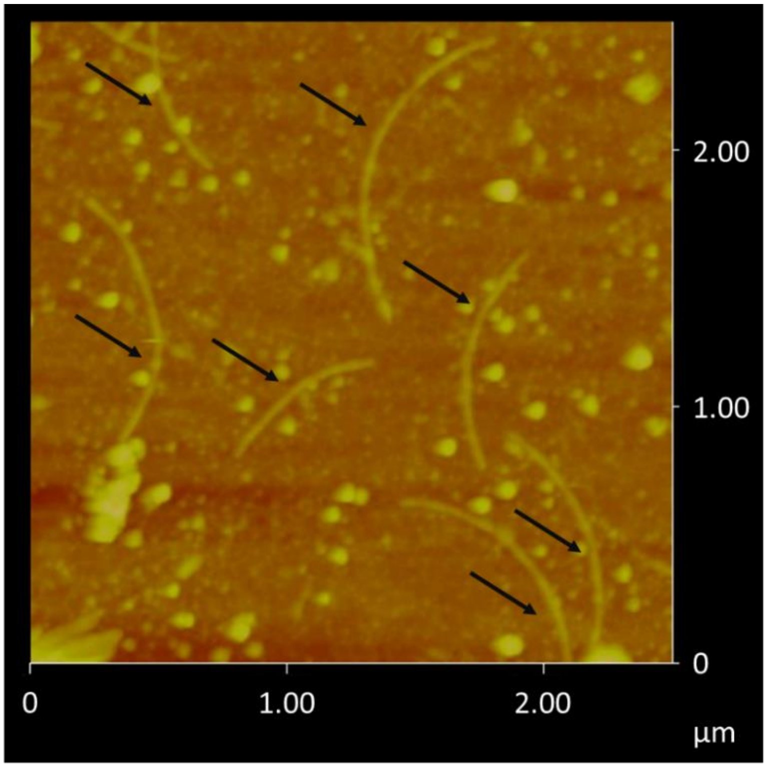

Supplemental Figure S8: AFM topography image of an unpurified OMV suspension. Several flagella are visible, marked with black arrows. Pili and proteins are also present though these cell debris are indistinguishable from OMVs in this image. The x- and y- scales are indicated in the image. The z-scale is from red to yellow with 0 to 75 nm respectively.

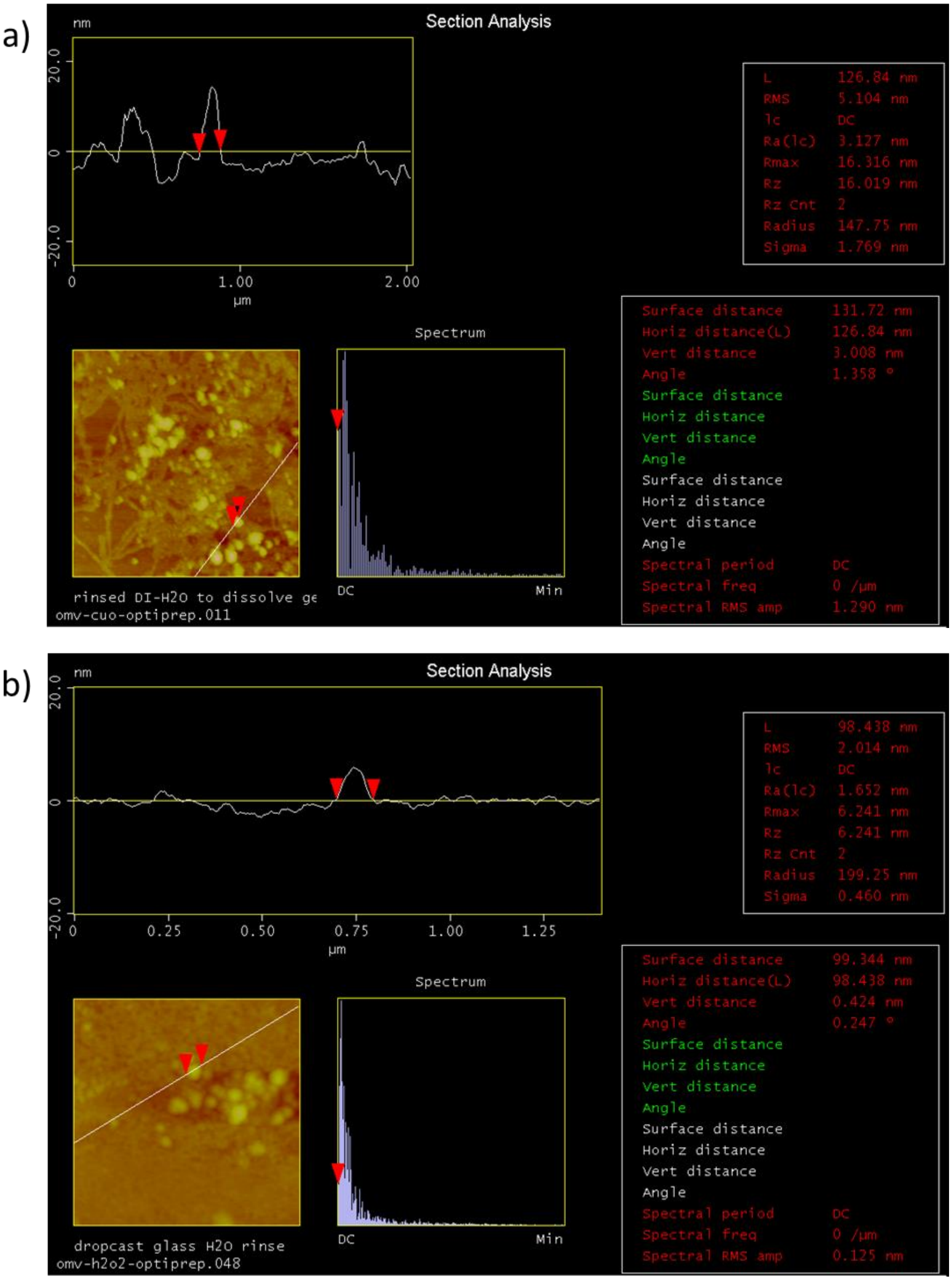

Supplemental Figure S9: Images showing AFM line cutting software used to determine OMV diameters in AFM images in a) CuO NP-induced OMVs and b) H_2O_2-induced OMVs.

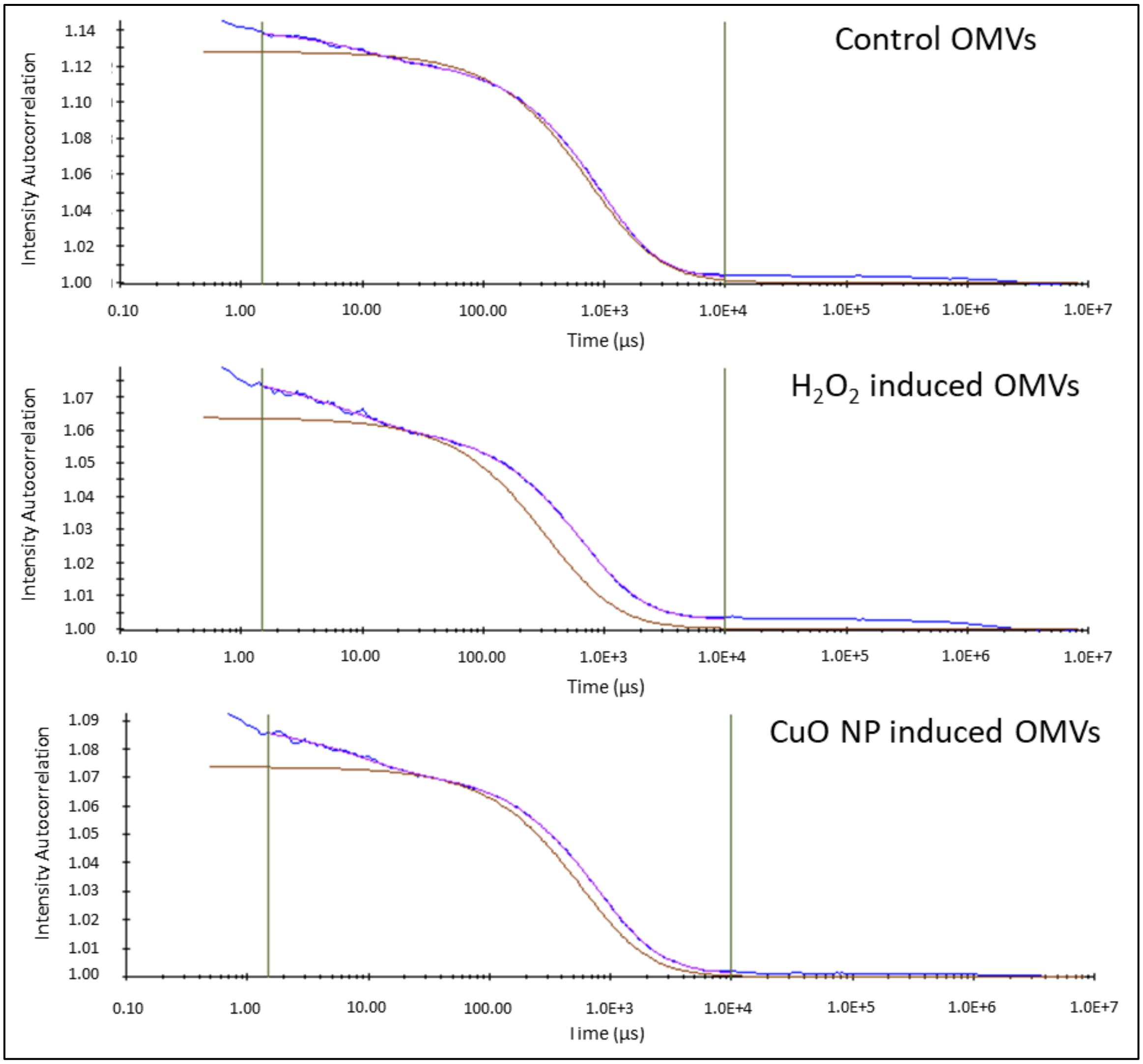

Supplemental Figure S10: Autocorrelation graphs given by DLS during measurements of OMV size.
